# A cryptic-site ligand stabilizes a non-canonical interface and blocks membrane insertion of the chloride intracellular channel CLIC1

**DOI:** 10.1016/j.jbc.2026.113113

**Published:** 2026-05-06

**Authors:** Shobhan Kuila, Archita Ghoshal, Sibasis Sahoo, Muthusankar Aathi, Mohd Azeem Khan, Love Panchariya, Kirti Shila Sonkar, Wajahat Ali Khan, Jaswanth Raj Pandiramesh, Anmol Chandele, Arockiasamy Arulandu

**Affiliations:** 1 Structural Biology Group, International Centre for Genetic Engineering and Biotechnology, New Delhi, India; 2 ICGEB-Emory Vaccine Centre, International Centre for Genetic Engineering and Biotechnology, New Delhi, India

**Keywords:** HsCLIC1, colorectal cancer, cryptic site, crystal structure, drug discovery, inhibitor, protein translocation

## Abstract

Human chloride intracellular channel 1 (HsCLIC1) is a dimorphic protein that exists in both soluble enzymatic and membrane-integrated ion channel forms. It is overexpressed in many cancers, yet developing specific inhibitors has been challenging, leaving CLIC proteins largely untargeted. Here, we identified a non-canonical ligand binding site on the soluble enzymatic form of HsCLIC1 and demonstrate that it is druggable with the small molecule inhibitor NSC602247. This cryptic site was discovered using multi-solvent molecular dynamics (MD) simulations followed by virtual screening and molecular docking. Using surface plasmon resonance and microscale thermophoresis, we show that NSC602247 binds to HsCLIC1 with micromolar affinity (∼6 μM) and inhibits the proliferation of HT29 colorectal cancer cells. To understand the mechanism of inhibition, we determined the crystal structure of the HsCLIC1-NSC602247 complex. Mechanistically, NSC602247 binding induces oligomerization of soluble HsCLIC1 and reduces its membrane expression, as revealed using flow cytometry. Our data clearly indicate that NSC602247 inhibits HT29 growth by sequestering the protein in a soluble state, thereby blocking its membrane translocation. These findings validate a novel cryptic site on CLIC1 and present a new mechanistic paradigm for inhibiting its pathological function by preventing membrane insertion, offering a promising strategy for targeted therapeutic development.

The human chloride intracellular channel (HsCLIC) family comprises six highly conserved proteins (HsCLIC1-6) that exhibit unique dimorphic properties, existing as both soluble cytoplasmic enzymes and membrane-integrated ion channels (1, 2, 3, 4, 5, 6).

HsCLIC1, an extensively studied family member, functions as a glutathione-dependent dehydroascorbate (DHA) reductase in its soluble form, playing a critical role in vitamin C recycling and cellular antioxidant defense (7, 8, 9). Under conditions of oxidative stress, increased intracellular pH, or changes in the membrane potential, HsCLIC1 undergoes a dramatic conformational transition, where it inserts into cellular membranes to form functional ion channels (10, 11, 12, 13, 14, 15, 16). As membrane-inserted channels, HsCLICs conduct ions with no apparent specificity. This redox-sensitive membrane insertion is mediated by the oxidation of cysteine residues, particularly Cys24, which triggers structural rearrangements that expose hydrophobic transmembrane domains (17). HsCLICs are overexpressed in a plethora of cancers, including breast ductal carcinoma (18), ovarian cancer, gastric cancer (19, 20, 21), nasopharyngeal carcinoma, hepatocellular carcinoma (22), and gliomas (23). HsCLICs are also known to play crucial roles in the cell cycle, apoptosis, proliferation, invasion, metastasis (24, 25, 26, 27, 28, 29), and cardio protection (30).

Furthermore, HsCLIC1 is considered a potential biomarker for the prognosis of metastasis in ovarian and oral squamous cancers (31, 32). HsCLIC1 is expressed at higher levels in colorectal cancer than in other types of cancer, as shown by the Cancer Genome Atlas dataset (Fig. S1). HsCLIC1 promotes colon cancer metastasis by facilitating cell migration and invasion through its chloride channel activity. Inhibiting HsCLIC1 with IAA-94, a known chloride channel inhibitor, or by translational knockdown inhibits colon cancer cell migration and invasion (33, 34). Reactive oxygen species (ROS) upregulate HsCLIC1 and drive its translocation to the membrane, further increasing ROS levels and metastatic potential (35). This ROS-HsCLIC1 feedback loop is key to the invasive traits of colon cancer cells and may operate similarly in other cancers under oxidative stress (36, 37). This crosstalk between ROS and HsCLIC1 could also be extended to other cancer cells, as they are under high oxidative stress. HsCLIC1 translocation from the cytoplasm to the membrane is known to be regulated by ROS and pH (38, 39, 40), and changes in ROS levels are fundamental for cell cycle progression (41, 42). Thus, the membrane expression of HsCLIC1 is directly related to cell cycle progression. Moreover, HsCLIC1 membrane insertion in cancer cells, especially in colorectal cells, appears to be regulated by signals such as ROS, which is consistent with its antioxidant enzyme activity. An expanded pool of cytoplasmic HsCLIC1 that undergo structural transition could facilitate tumor progression, which is characterized by a high propagation rate and invasion (43, 44, 45). Collectively, these data indicate that HsCLIC1 is essential for cancer cell survival and represents a compelling molecular target for structure-based drug discovery and development (46, 47).

Despite compelling evidence for the therapeutic potential of HsCLIC1, the greatest challenge in targeting HsCLIC1 is that the canonical substrate binding sites (G- and H-sites) exhibit poor druggability, characterized by high polarity and narrow binding clefts (9) (Fig. 1*A*). Furthermore, the active site of CLIC1 is conserved among GST family members, suggesting possible selectivity concerns and off-target effects. Furthermore, traditional chloride ion channel inhibitors such as IAA-94 and DIDs (4,4′-diisothiocyanatostilbene-2,2′-disulfonic acid) lack specificity and exhibit significant toxicity, limiting their therapeutic utility (48, 49). The dynamic nature of HsCLIC1, with its ability to transition between soluble and membrane-bound states, presents both challenges and opportunities for drug design. Targeting the soluble form to prevent pathological membrane insertion while preserving essential enzymatic functions, potentially offers superior therapeutic windows compared with direct channel blockade.

**Figure 1:**
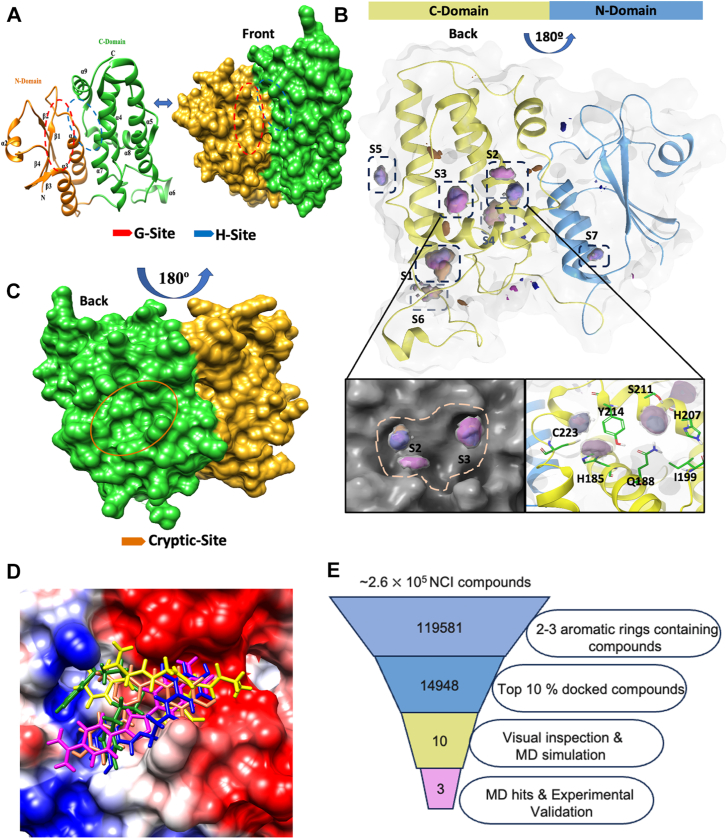
**Discovery and targeting the non-canonical ligand-binding site on HsCLIC1.***A*, CLIC1 with two-domain architecture comprising an N-terminal domain (*gold*) and a C-terminal domain (*green*). The surface view highlights the conserved GST-like active site, with the G site indicated by a red circle and the H-site indicated by a *blue circle*. *B*, seven druggable cryptic sites labeled S1-S7 are shown on the HsCLIC1 surface. The key residues from these sites; H185, Q188, I199, H207, S211, Y214, and C223, that were used to build the grid for molecular docking are shown in the inset. *C*, the cryptic site on HsCLIC1 is shown. *D*, electrostatic surface of CLIC1 showing docked small molecules at the cryptic site. *E*, schematic representation of the virtual screening workflow used to identify small-molecule binders of CLIC1.

In this study, we employed an integrated structure-based approach to overcome these challenges. We sought to identify a novel, druggable cryptic site on soluble HsCLIC1, discover small molecules that target this site, and elucidate the mechanism of inhibition through biophysical methods and high-resolution structural biology. Finally, we aimed to validate the functional consequences of this inhibition on cell viability and CLIC1 membrane translocation in a cell-based colorectal cancer model.

## Results

### Identification and targeting of a non-canonical ligand binding site in HsCLIC1

To identify druggable pockets beyond the canonical G- and H-sites, we performed mixed-solvent molecular dynamics (MixMD) simulations on the high-resolution structure of soluble HsCLIC1 (50) (Fig. 1*B*, PDB: 1K0M). This approach utilized multiple organic probes (acetonitrile, isopropanol, and pyrimidine) to explore transient pocket formation, solvent occupancy and residence time distributions by delineating regions with a high propensity for small-molecule binding.

Seven distinct hotspots, S1-S7, were identified across the HsCLIC1 surface, each representing a potential binding site. The hotspots, S1-S3, form a contiguous, non-canonical binding region in the C-terminal with a combined volume above the commonly accepted druggability threshold (greater than ∼250 Å^3^ for composite clusters; individual local maxima exceeding 45 Å^3^), featuring a hydrophobic core flanked by hydrogen bond donors/acceptors (Fig. 1*B*). This site is composed primarily of residues Y214, C223, H185, Q188, I199, H207, and S211 and presents complementary aromatic and polar features suitable for π-stacking and directional H-bonding. Comparative analysis of existing HsCLIC1 crystal structures revealed that this cryptic site is solvent exposed in the soluble monomeric, pre-insertion state but is occluded upon oligomerization and/or membrane insertion. This led us to target this site with high-affinity binders to shift the conformational equilibrium toward the pre-inserted soluble state.

To identify molecules that specifically bind to the non-canonical site in the C-terminal domain, we performed virtual screening of 267,000 small molecules from the National Cancer Institute Enhanced database. MixMD solvent mapping indicated that site recognition would favor ligands with aromatic surface area and moderate lipophilicity. Accordingly, a cheminformatics filter retained scaffolds containing two to three aromatic rings to enable π−σ and π−π interactions with residues Y214 and H207 and logP values between 1 and 4 to balance hydrophobic enclosure with aqueous compatibility. This reduced the library to 119,581 candidates (Fig. 1*E*) for high-throughput docking (Glide, Schrödinger). The top-ranked poses were rescored using MM-GBSA to incorporate local protein flexibility and solvation contributions, yielding a prioritized set of chemotypes.

The top 10% of the docked compounds were clustered on the basis of their scaffold and manually inspected for key interactions with the non-canonical cryptic site (Fig. 1*D*). Ten compounds were shortlisted for molecular dynamics (MD) simulation on the basis of their docking score (−6.8 to −8.9 kcal/mol) and MM-GBSA binding free energy (−20.0 to −45.2 kcal/mol). Three compounds (NSC240900, NSC602247, and NSC51648) showed stable binding with favorable MM-GBSA profiles and recurrent engagement of the aromatic hotspot around Y214. Among these, Y214 consistently contributed ∼7.5 to 8.5 kcal/mol to the binding energy across trajectories, highlighting a convergent recognition motif.

**Figure 2:**
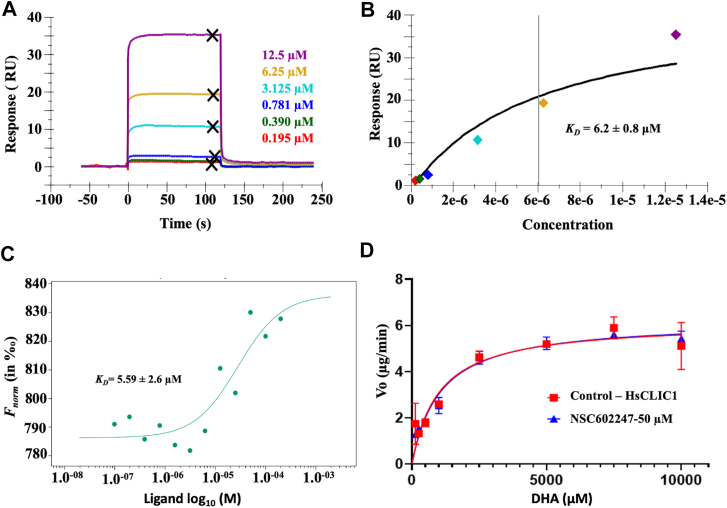
**Biophysical characterization of NSC602247 binding to CLIC1.***A*, SPR multicycle kinetics of NSC602247 with HsCLIC1 is shown. Various concentrations of NSC602247 from 12.5 μM to 195 nM were passed over CLIC1 immobilized on a CM5 chip. The change in response at the end of each association event (*black x mark*) is plotted against the ligand concentration and *B*) Data fitted into steady-state kinetics is shown. The calculated affinity (*K*_*D*_) was 6.2 ± 0.8 μM. *C*, the fitted microscale thermophoresis data for different concentrations of NSC602247 (200 μM to 97.56 nM) incubated with fluorescently labeled HsCLIC1 for 10 min. Normalized fluorescence (Fnorm) is plotted against the concentration of ligand. The affinity was 5.59 ± 2.6 μM, as determined by fitting the data into the KD Model. *D*, enzyme inhibition assay: the rate of formation of Ascorbate [Vo (μg/min)] in the absence or presence of the small molecule NSC602247. For the enzyme inhibition assay, 50 μM NSC602247 (*blue line*) was added to the enzyme mixture with increasing concentrations of the substrate dehydroascorbate, and the results were compared with those of the control (*black line*). Vo (μg/min) denotes the rate of formation of ascorbate at different concentrations of dehydroascorbate. Both the control and the treatment groups showed no significant changes.

To experimentally validate the binding, we performed surface plasmon resonance (SPR) with purified CLIC1 and MD-validated hits. Among the three MD-stable hits, NSC602247 showed a clear concentration-dependent SPR response and followed 1:1 steady-state kinetics. In contrast, NSC240900 and NSC51648 produced weak or noisy signals and non-ideal sensorgrams that did not indicate specific 1:1 binding under the assay conditions. Multicycle kinetics analysis of SPR with NSC602247 yielded a *K*_*D*_ of 6.2 ± 0.8 μM (Fig. 2, *A* and *B*), which was confirmed by microscale thermophoresis (MST) measurements, which revealed a similar *K*_*D*_ = 5.59 ± 2.6 μM (Fig. 2*C*). This compound was further pursued in biochemical, structural, and cell-based studies.

### NSC602247 does not alter enzyme activity

To determine whether this binding alters enzymatic function, we performed a competitive enzyme assay using a fixed concentration of NSC602247 (50 μM) and varying concentrations of the DHA substrate. The reaction kinetics revealed no difference in the V_max_ between the control (6.140 μg/min) and NSC602247-treated samples (6.218 μg/min). Similarly, the K_m_ values remained comparable between the control (986.9 μM) and treated (1049 μM) conditions (Fig. 2*D*). These results indicate that NSC602247 binds to a site distal to the active site of HsCLIC1 and does not measurably alter its enzymatic activity, which is consistent with the above docking analysis.

Taken together, these findings establish that NSC602247 binds specifically and with single digit micromolar affinity to HsCLIC1, providing a mechanistic basis for subsequent structural and functional characterization.

### Crystal structure reveals a ligand-stabilized cryptic interface

**Figure 3:**
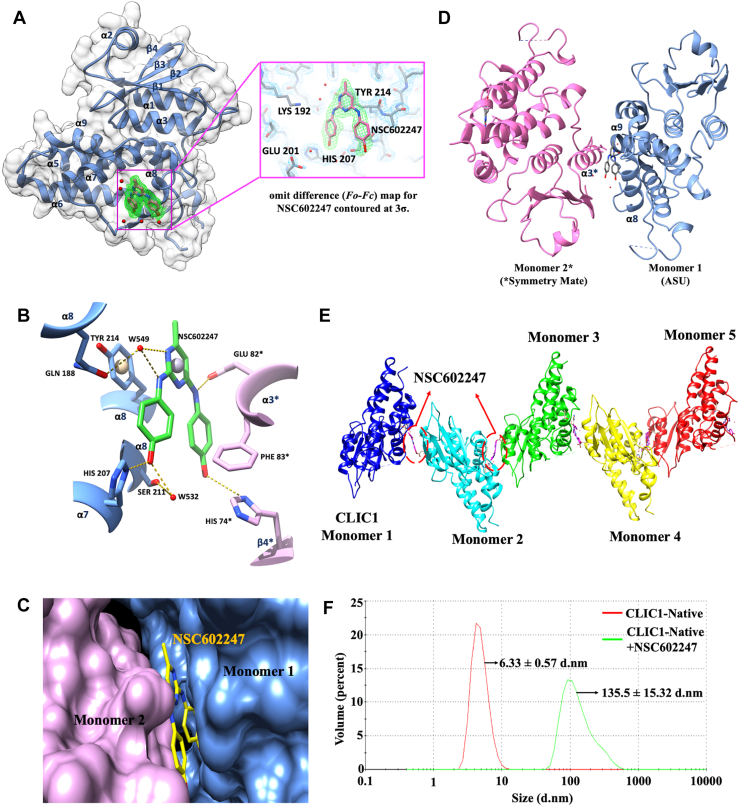
**Crystal structure of the CLIC1-NSC602247 complex (PDB:7FBQ).***A*, NSC602247 is bound at the non-canonical site on HsCLIC1. The electron density for the ligand (*inset*) is shown in the omit difference (Fo–Fc) map contoured at 3σ within the ASU. *B*, NSC602247 makes direct contact with two CLIC1 monomers, bringing the molecules together through Gln188, His207, Ser211, and Tyr214 in chain A (ASU), and His74∗, Glu82∗, and Phe83∗ in chain B∗ (∗symmetry mate). Water-mediated interactions involve ordered water molecules W532 and W549. *C*, surface representation of the dimer interface with bound NSC602247. *D*, NSC602247 binds at the interfacial region between two CLIC1 monomers, stabilizing the dimeric form of HsCLIC1. *E*, crystal packing of HsCLIC1 shows a higher-order oligomeric arrangement induced by NSC602247 binding at the dimer interface. *F*, dynamic light scattering analysis of HsCLIC1 in solution. The *green* trace represents apo-CLIC1 with a Z-average diameter of 6.33 ± 0.57 nm. The *red trace* shows a shift to larger particle size, with a Z-average diameter of 135.5 ± 15.32 nm, upon addition of NSC602247.

NSC602247 markedly enhanced the crystallization behavior of HsCLIC1, yielding well-ordered crystals within minutes across multiple buffer conditions. The best crystals were obtained in 20% PEG 6000, 0.2 M MgCl_2_, and 100 mM MES, pH 6.0, with 25 mg/ml HsCLIC1 and 5 mM ligand. These crystals diffracted to 1.6 Å resolution and belonged to the orthorhombic space group P2_1_2_1_2_1_, containing one monomer per asymmetric unit, and a solvent content of 42.1%. Clear *Fo-Fc* electron density for NSC602247 was observed at the predicted cryptic site, and ligand fitting achieved a real-space correlation coefficient of 0.92 (Fig. 3*A* and Table 1). The final model was refined to *R*_*work*_*/R*_*free*_ values of 0.18/0.21 (PDB: 7FBQ).

**Table 1:** Data collection and refinement statistics of X-ray crystallography

Parameters	CLIC1-NSC602247 (PDB:7FBQ)	CLIC1-Y214A (PDB:24UG)
ASU	One monomer	Two Monomers
Data collection		
Space group	P2_1_2_1_2_1_	P2_1_
Cell dimensions		
*a, b, c* (Å)	42.00, 67.34, 81.38	41.94, 68.39, 81.75
*α, β, γ* (°)	90, 90, 90	90, 90.09, 90
Resolution (Å)	19.65–1.80 (1.83–1.80)	29.25–2.50 (2.60–2.50)
*R*_*merge*_	0.087 (0.679)	0.123 (0.247)
*I/σI*	18.6 (3.5)	12.9 (6.7)
Completeness (%)	94.5 (84.4)	99.8 (100)
Redundancy	12.6 (11.4)	9.4 (9.4)
*CC*_1/2_	0.99 (0.88)	0.99 (0.97)
Refinement		
Resolution (Å)	19.65–1.80	29.25–2.50
No. reflections	20,809	15,758
*R*_*work*_/*R*_*free*_	0.183/0.207	0.182/0.240
No. atoms		
Protein	1847	3911
Ligand/ion	23	-
Water	258	244
B-factors (Å^2^)		
Protein	25	27
Ligand (N70)	19.5	-
r.m.s. deviations		
Bond lengths (Å)	0.008	0.003
Bond angles (°)	0.85	0.571
Ligand real-space correlation coefficient		
NSC602247 (PDB:N70)	0.92	-
CSD	0.96	-

Values in parentheses are for the highest-resolution shell.

Structural superposition of NSC602247-bound HsCLIC1 with apo HsCLIC1 (PDB: 1K0M) revealed no global conformational changes apart from a shift in the human-specific CLIC1 loop (P147 to L173). The active site (G- and H-sites) and N-terminal domain remained unaltered, and the catalytic cysteine was found in an oxidized sulfinic (–SO_2_H) state, likely due to crystallization artifacts (Fig. S2). NSC602247 occupies a cryptic binding site at the interface between two HsCLIC1 monomers. Within the asymmetric unit, the ligand contacts Tyr214, Gln188, His207, and Ser211 of one monomer while also engaging His74, Glu82 and Phe83 from the adjacent symmetry-related monomer (Fig. 3*B*). This interface is mediated by α-helices 8 and 9 from one monomer and α-helix 3 from the symmetry mate, which are stabilized further by two bridging water molecules (W532, W549).

The nitrogen and oxygen atoms of NSC602247 contribute to the precise orientation of the ligand in the binding pocket. Specifically, the two oxygen atoms of NSC602247 (O and O1 atoms) form direct hydrogen bonds with the delta nitrogen atom (ND1 atom) of the His207 side chain and water-mediated hydrogen bonds with Ser211 through W532 (HOH) (Fig. 3*B*). The aniline nitrogen (N2) that bridges the two six-membered aromatic rings establishes a water-mediated hydrogen bond with Gln188 through W549. In the symmetry-related monomer, the second aniline nitrogen atom (N3) of the ligand forms a hydrogen bond with Glu82 (OE1), whereas the Phe83 benzene ring generates a hydrophobic cavity that accommodates the ligand’s benzene ring, enabling additional flexible π–π stacking interactions (Fig. 3*B*). The ligand-mediated monomer–monomer interface is distinct from those reported in other HsCLIC1 crystal structures available in the Protein Data Bank, burying a surface area of 298 Å^2^ with an interface free energy (ΔG) of −2.8 kcal/mol. This unique interface, stabilized by NSC602247, underscores its role in locking the protein in the soluble form while revealing the cryptic binding site.

The binding of NSC602247 induces the formation of a tight pocket at the interface of two HsCLIC1 monomers, resulting in a compact dimeric arrangement, as revealed by the crystal structure of the HsCLIC1–NSC602247 complex (Fig. 3, *C* and *D*). In the higher-order assembly observed in the crystal lattice (PDB: 7FBQ), each NSC602247 molecule simultaneously occupies Site 1 of one monomer and Site 2 of the adjacent symmetry-related monomer, thereby bridging successive monomers in the chain (Fig. 3*E*), with a binding stoichiometry of one NSC602247 molecule per monomer.

Notably, during the *in silico* screening and docking workflow, NSC602247 was docked onto a single HsCLIC1 monomer, specifically monomer 1 (colored blue; Fig. 3*D*), prior to crystal structure determination. The predicted binding pose occupies the same intra-monomer cryptic pocket (Site 1: Gln188, His207, Ser211, Tyr214, Fig. S4*A*) observed in the co-crystal structure. Superposition of the docked and crystallographic poses (Fig. S3) shows that, although the ligand occupies the same site, it adopts a slightly different orientation in the crystal structure, likely reflecting the influence of the adjacent symmetry-related monomer (monomer 2, colored pink) on the final binding geometry, an interaction not captured in the single-monomer docking model. Importantly, the crystal structure further reveals that NSC602247 simultaneously engages a second binding site (Site 2: His74, Glu82, Phe83) on monomer 2 (Figs. 3*D* and S4*B*). This Site 2 interaction was not predicted by docking and is observed only in the dimeric assembly, exhibiting a substantially weaker predicted binding affinity compared to Site 1.

Having established the structural basis of NSC602247 binding at the cryptic interfacial site, we next investigated whether this ligand-induced dimerization observed in the crystal lattice extends to higher-order oligomeric assembly in solution.

### NSC602247 induces higher-order oligomerization of HsCLIC1 in solution

To further examine whether NSC602247 also promotes oligomerization in solution, dynamic light scattering (DLS) experiments were conducted. The addition of NSC602247 led to a marked increase in the hydrodynamic diameter (d.nm), with a Z-average of 135.5 ± 15.32 nm, compared with the ∼6.33 ± 0.57 nm observed for reduced apo HsCLIC1, which reflects a predominantly monomeric species (Fig. 3*F*). These results indicate that NSC602247 drives the formation of higher-order oligomers in solution. On the basis of DLS data and crystal structure analysis, we hypothesize that NSC602247 binds at two unique sites on two HsCLIC1 monomers, leading to chain formation and small aggregates in solution, which are dependent on the amount of ligand added.

Notably, ligand-induced oligomerization is structurally distinct from previously reported HsCLIC1 dimers. A comparison of the NSC602247-bound dimer (PDB: 7FBQ) with the oxidized dimer (PDB: 1RK4; (11)) revealed diverse interfaces and domain arrangements. In the oxidation-induced dimer, α-helices 2 and 3 of one monomer contact α-helices 3 and 2 of the partner, occluding the active site. In contrast, NSC602247 binding promotes an alternative dimerization mode in which α-helices 8 and 9 of one monomer engage α-helix 3 of the other, leaving the active site accessible and conformationally unperturbed (Fig. S5, *A* and *B*).

Structural superposition revealed that the active site remains intact in the NSC602247-bound complex (PDB: 7FBQ), whereas the oxidized dimer (PDB: 1RK4) substantially rearranged the N-terminal domain into an all-helical fold that compromises access to the active site (Fig. S5*C*). Together, these findings suggest that NSC602247 stabilizes a cryptic interfacial pocket and enforces a distinct oligomerization pattern that preserves the catalytic architecture of HsCLIC1 while promoting its assembly into higher-order species in solution.

### NSC602247 stabilizes a compact HsCLIC1 dimer through an interfacial aromatic hotspot

**Figure 4:**
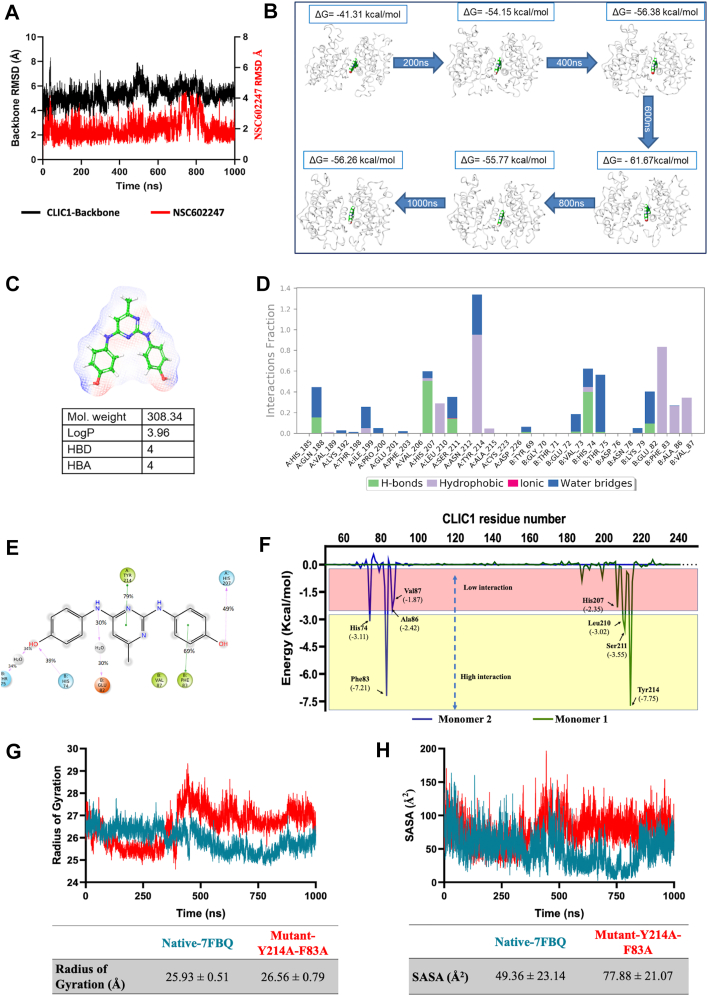
***In silico* analysis of the CLIC1-NSC602247 interaction.***A*, Root mean square deviation (RMSD) during a 1 μs molecular dynamics (MD) simulation showing the ligand (*red*) and the CLIC1 backbone (*black*), with average RMSD values of 5.3 ± 0.6 Å and 2.0 ± 0.6 Å, respectively. *B*, ΔG changes during the course of 1000 ns of MD are shown. *C*, physicochemical properties of the ligand NSC602247. *D*, residues and molecular forces involved in CLIC1- NSC602247 complex formation are shown. *E*, Time, in %; during which the intermolecular interaction remains stable throughout the 1000 ns, as assessed *via* MD simulation. *F*, residue-wise contribution to the total ΔG change upon interaction with NSC602247 from both chains of the CLIC1 dimer. *G*, radius of gyration of the HsCLIC1-NSC602247 complex and *H,* solvent accessible surface area (SASA) of the ligand binding site with and without mutation of Y214A and F83A∗. ∗From the symmetry related monomer 2.

To assess the stability and dynamics of the HsCLIC1-NSC602247 dimeric complex (PDB: 7FBQ), a 1000 ns all-atom MD simulation was performed. NSC602247 remained stably bound at the interface pocket throughout the trajectory. The root mean square deviation (RMSD) relative to the initial complex indicated a limited structural drift, with values of 5.3 ± 0.6 Å for the HsCLIC1 backbone and 2.0 ± 0.6 Å for NSC602247 (Fig. 4*A*). Consistent with the packing of the interface, the estimated binding free energy (ΔG) became more favorable during the simulation, increasing from −41.31 to −56.26 kcal/mol over 1 μs, in parallel with increased compaction, indicating an energetically stabilized HsCLIC1-NSC602247 complex (Fig. 4*B*).

Interface analysis revealed a partially hydrophobic pocket spanning the C-terminal region of monomer 1 (Leu210, Tyr214) and the N-terminal region of monomer 2 (Val73, Phe83, Ala86, Val87). The aromatic scaffold of NSC602247 resides within this cavity and forms dominant π–π contacts with Tyr214 (Site 1) and Phe83 (Site 2). In addition, recurrent hydrogen bonds were observed with His185, His207, Ser211, Val73∗, and His74∗ (∗ denotes Site 2 residues). Structured water further stabilized the complex by mediating water-bridged interactions with His185, Ser211, Thr75, and Glu82∗; these contacts persisted dynamically with varying occupancies over the trajectory (Fig. 4, *D*–*F*).

Ligand binding is associated with compaction of the dimeric assembly. Visual inspection and radius of gyration (R_g_) analysis revealed a decrease from ∼27 Å at the beginning to 25.93 ± 0.51 Å, indicating reduced intermonomer separation and a more compact quaternary structure (Fig. 4, *G* and *H*). To test the functional relevance of the aromatic hotspot, Tyr214 (Site 1) and Phe83 (Site 2) were mutated to alanine *in silico*, followed by MD simulations in the presence of NSC602247. In contrast to the native complex, the double mutant failed to maintain ligand binding, and the dimer dissociated as the simulation progressed. Correspondingly, the R_g_ and solvent accessible surface area increased sharply in the mutant simulations (Fig. 4, *G* and *H*), supporting a critical role for Tyr214 and Phe83∗ in ligand-mediated dimer stabilization.

Further, to experimentally confirm the role of Tyr214 in ligand binding, we substituted this residue with alanine and conducted biophysical and structural characterization. First, we performed MST measurements, which showed a significant impairment in ligand binding across 97.5 nM to 200 μM ligand concentrations (Fig. S6), while the native CLIC1 exhibited a robust, concentration-dependent change in fluorescence upon titration with NSC602247 and *K*_*D*_ determination (Fig. 2*C*). Consistent with this loss of interaction, DLS analysis showed no ligand-induced aggregation in the Y214A variant with 200 μM of NSC602247 (Fig. S7), further supporting the absence of productive ligand engagement. Additionally, the crystal structure of the CLIC1 Y214A mutant (PDB: 24UG), obtained under identical conditions as the native protein, revealed no electron density for NSC602247 (Fig. S8).

Collectively, these results indicate that NSC602247 forms a stable, well-packed interfacial complex with HsCLIC1 that is reinforced by π–π interactions at the aromatic hotspot and auxiliary hydrogen bonds, including water-bridged contacts. Ligand binding promotes dimer compaction, whereas disruption of the hotspot abrogates binding and destabilizes dimeric assembly. Therefore, we hypothesize that NSC602247 binding stabilizes a soluble, dimeric conformational state of HsCLIC1 and shifts the conformational equilibrium away from membrane-competent intermediates, thereby preventing membrane insertion and subsequent channel formation under insertion-promoting conditions.

### NSC602247 impairs HT29 cell growth and inhibits HsCLIC1 membrane translocation

**Figure 5:**
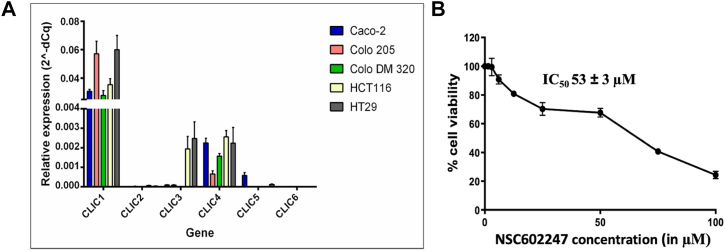
***CLIC1* expression in various colon cancer cell lines and cell viability assay.***A*, *CLIC1* transcript levels in the cell lines Caco-2, Colo-205, Colo DM-320, HCT116, and HT29 were assessed *via* qRT‒PCR. *B*, cell viability assay with NSC602247 in the HT29 cells showed an IC_50_ of 53 ± 3 mM (n = 3). Statistical analyses (one-way ANOVAs) were conducted in GraphPad Prism, with Dunnett’s *post hoc* tests for comparisons. “∗∗∗∗” signifies *p* < 0.0001.

**Figure 6:**
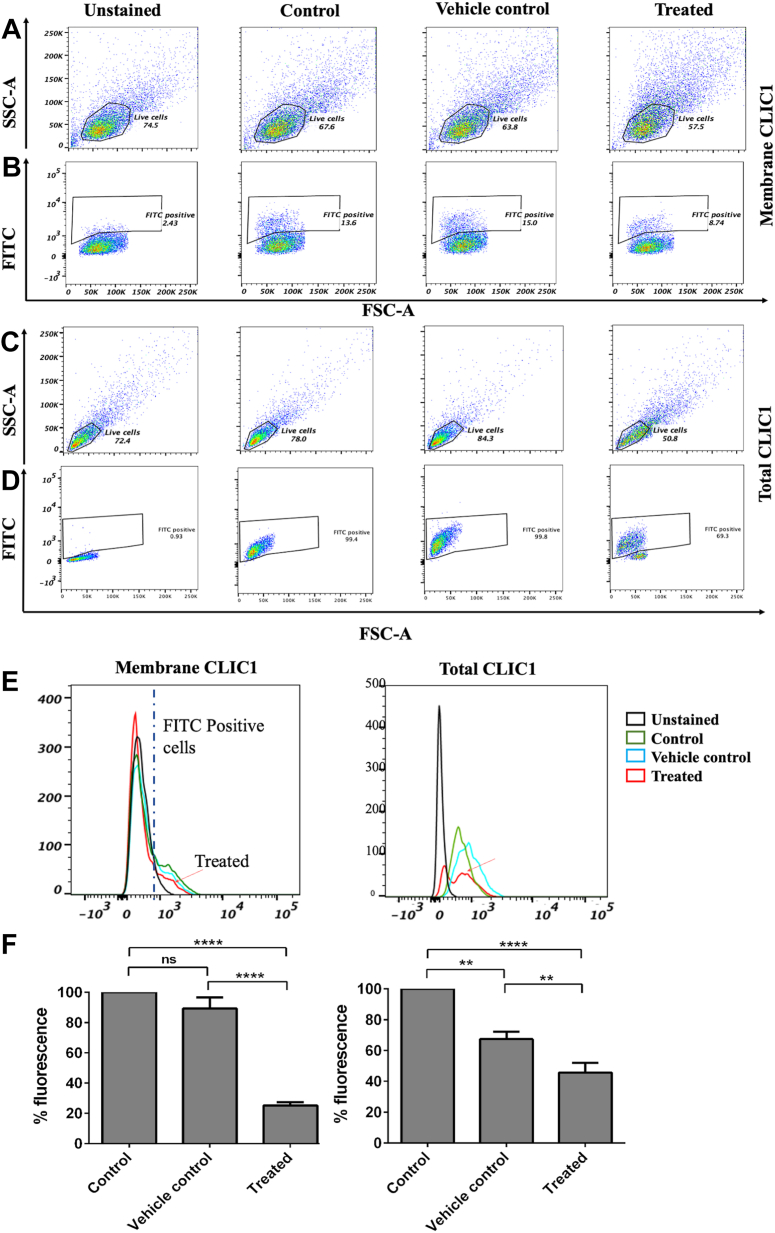
**NSC602247 inhibits the surface expression of CLIC1 in HT29 cells.** Assessment of the expression of surface and total HsCLIC1 in HT29 cells using flow cytometry revealed a significant reduction in membrane CLIC1 expression after 48 h of treatment with 10 μM NSC602247. The control represents untreated cells; the vehicle control represents cells treated with 0.01% DMSO equivalent to the DMSO concentration in treated cells. *A*, percentage of live cells gated while probing for membrane expression. *B*, Percentage of cells positive for membrane HsCLIC1 staining. *C*, total CLIC1 staining. *D*, percentage of cells positive for total CLIC1 expression. *E*, histograms show membrane (*left panel*) and total (*right panel*) CLIC1 staining. *F*, graphical representation of CLIC1 expression shown in histograms of % fluorescence.Statistical analyses (one-way ANOVAs) were conducted in GraphPad Prism, with Tukey’s *post hoc* tests for comparisons. “∗∗∗∗” signifies *p* < 0.0001.

First, we profiled the expression of all six human CLIC proteins (HsCLIC1-6) across colorectal cancer cell lines: Caco-2, Colo-205, Colo DM 320, HCT116, and HT29, and selected HT29 for downstream assays on the basis of its highest HsCLIC1 expression (Fig. 5*A*). NSC602247 reduced HT29 viability in a concentration-dependent manner, with an IC50 of 53 ± 3 μM (Fig. 5*B*). Furthermore, HT29 cells were stained with fluorescently labeled anti-HsCLIC1 antibodies (51) and analyzed by flow cytometry. NSC602247 treatment significantly decreased both the membrane-associated and total (membrane and cytoplasmic) HsCLIC1 signals. Consistent with prior reports that HsCLIC1 translocates to the plasma membrane under specific stimuli, untreated cells presented moderate membrane HsCLIC1, whereas NSC602247 abrogated membrane staining, reducing the mean and median fluorescence intensities to near-unstained levels (Fig. 6, *A*–*F*).

**Figure 7:**
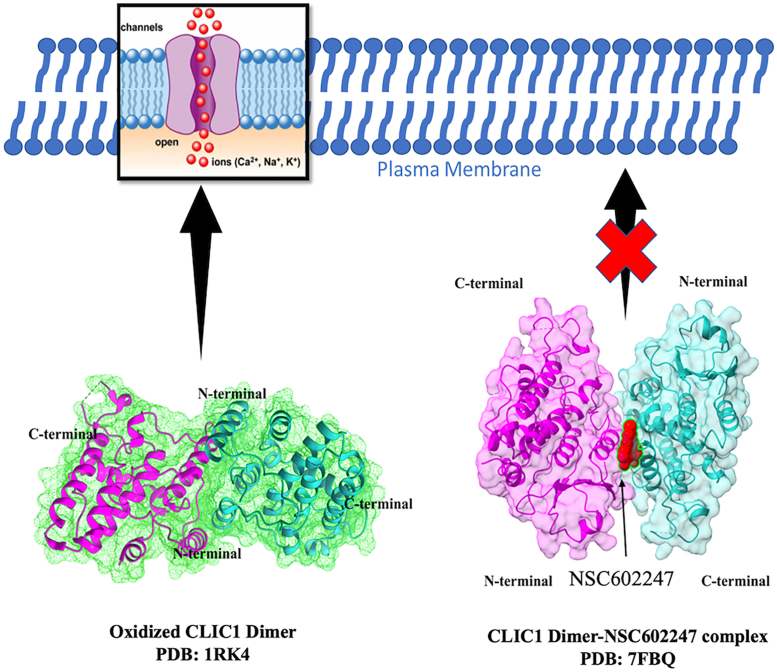
**Model for the inhibition of CLIC1 membrane insertion by NSC602247.** non-canonical dimerization by NSC602247 hinders CLIC membrane insertion, affecting cancer cell progression.

These observations indicate that NSC602247 inhibits HsCLIC1 membrane translocation in HT29 cells, an effect consistent with the ligand-induced stabilization of soluble oligomeric assemblies observed in our structural and biophysical studies. Notably, NSC602247 also decreased the total HsCLIC1 signal detected by flow cytometry; while this could reflect reduced protein abundance, it may also arise from ligand-induced compaction and higher-order oligomerization of HsCLIC1 masking the epitopes recognized by the anti-HsCLIC1 polyclonal antibodies, an interpretation supported by our *in vitro* DLS data demonstrating higher-order assembly upon ligand addition (Fig. 3*F*). Together, these cellular observations are consistent with a model in which NSC602247 sequesters HsCLIC1 in a soluble, non-membrane-insertion-competent state, thereby impairing its translocation to the plasma membrane (Fig. 7). However, direct biochemical confirmation of on-target cellular engagement remains to be established in future studies.

## Discussion

This study provides a new strategy for CLIC1 drug discovery by identifying and validating the druggable cryptic site on human CLIC1, addressing longstanding challenges in targeting this clinically relevant protein. Through a combination of structure-based drug design, virtual screening, and both structural and *in vitro* validation, we demonstrate that allosteric modulation of the conformational equilibrium of CLIC1 provides an effective alternative to traditional active site inhibition, yielding enhanced selectivity while preserving normal physiological function.

Our findings offer several key insights. First, MixMD simulations enabled the discovery of a cryptic, druggable pocket on soluble HsCLIC1 that is distinct from its canonical GSH-binding active site and accessible only in the monomeric, pre-membrane insertion state. Second, the crystal structure of the HsCLIC1-NSC602247 complex, supported by MD simulations, revealed a sophisticated allosteric mechanism of inhibition wherein ligand binding induces and stabilizes a compact dimeric assembly incompatible with membrane insertion but preserves enzymatic accessibility. The specificity of this mechanism is confirmed by *in silico* mutagenesis and experimental mutational studies: disruption of key aromatic hotspot residues (Tyr214, Phe83) abolishes both ligand binding and oligomer stability, providing a solid foundation for structure-activity optimization. Third, we demonstrate the potent antiproliferative effects of NSC602247 against HT29 colorectal cancer cells and the selective targeting of membrane-associated HsCLIC1, establishing a clear mechanism-of-action relationship in which reduced membrane CLIC1 expression aligns with growth inhibition. This selectivity suggests a favorable therapeutic window, as cancer cells show increased dependence on CLIC1-mediated functions for proliferation, invasion, and stress resistance, whereas normal cells, with lower CLIC1 expression, are relatively spared owing to diminished reliance on CLIC1-dependent pathways.

Mechanistically, our data show that NSC602247 binding induces and stabilizes a dimeric assembly through direct intermolecular contacts, effectively trapping CLIC1 in a soluble state that is not competent for membrane insertion. This inhibitor-induced dimerization is distinct from the oxidation-induced dimerization that occludes the active site, thus preserving the essential enzymatic functions of CLIC1 while blocking membrane insertion. Such functional selectivity is challenging to achieve with competitive inhibitors and addresses the limitations of broad-spectrum chloride channel blockers, which typically lack specificity and exhibit significant toxicity (52, 53).

In addition to its therapeutic potential, NSC602247 is a valuable chemical probe for dissecting CLIC1 biology. Its ability to trap CLIC1 in a defined conformational state provides a powerful tool for investigating the molecular mechanisms underlying CLIC1-driven pathogenesis and for validating CLIC1 as a therapeutic target across different cancer contexts.

The successful targeting of the CLIC1 cryptic site has broader implications for drug development against conformationally dynamic targets. Structural conservation within the CLIC family suggests that analogous cryptic sites may exist on CLIC2-6, allowing the development of pan-CLIC inhibitors or isoform-selective modulators through rational scaffold modifications. In general, this work highlights the value of targeting biologically relevant conformational transitions rather than static binding sites, a strategy relevant for allosteric enzymes, disordered proteins, and other challenging dynamic targets that are traditionally considered "undruggable".

Several limitations warrant consideration for future development. The micromolar potency of NSC602247 validates the druggability of HsCLIC1, however, medicinal chemistry optimization is essential to achieve clinically relevant potencies. Structure-guided design based on the cocrystal structure should focus on enhancing complementarity to the cryptic pocket, improving binding kinetics, and optimizing pharmacokinetic properties. Comprehensive selectivity profiling across CLIC paralogs and related GST family members will be essential to confirm target specificity and minimize off-target risks, given the conservation of certain structural features within these protein families. Additionally, while cell-based studies demonstrate reduced membrane CLIC1 expression, direct biochemical assays of membrane insertion under controlled redox, pH, and ionic conditions would provide more definitive mechanistic validation, and functional assays of channel activity in reconstituted systems would directly confirm that ligand binding prevents the formation of functional ion channels. Although direct cellular target engagement such as CETSA was not established in this study, the interaction between NSC602247 and HsCLIC1 is robustly supported by convergent structural and biophysical evidence, including the co-crystal structure confirming ligand occupancy at the cryptic site, SPR and MST binding measurements, and complete abolition of binding upon Y214A mutation. The absence of a thermal shift in TSA experiments (Fig. S9) is consistent with the moderate affinity (∼6 μM) and the shallow, ligand-induced interfacial nature of the binding site, which is inherently less amenable to enthalpy-driven stabilization. The cellular observations reported here are therefore best interpreted as consistent with the proposed mechanism, and establishing definitive cellular target engagement through orthogonal approaches remains an important goal for future studies. This study employs pharmacological inhibition rather than genetic depletion of HsCLIC1, a choice informed by well-documented functional compensation among CLIC family members. Multiple studies have demonstrated that single-gene loss or knockdown of CLIC1 is frequently compensated by other family members, particularly CLIC2 and CLIC4, resulting in mild, variable, or context-dependent phenotypes (6, 9, 23, 54, 55, 56), whereas combined depletion of multiple CLIC proteins produces more pronounced defects in processes such as cytokinesis, endothelial signaling, and vascular development (55, 56). Pharmacological inhibition by NSC602247 acutely perturbs CLIC1 membrane translocation on a timescale that precedes such compensatory adaptations (6, 9, 55, 56), thereby more directly capturing the immediate functional contribution of CLIC1 to cancer cell proliferation. Notably, residual compensation by other CLIC family members may also partly account for the moderate antiproliferative potency observed for NSC602247 (∼53 μM).

This promising *in vitro* proof-of-concept supports advancements toward preclinical development. Key priorities include lead optimization to improve potency and drug-like properties, comprehensive safety and toxicology studies, and *in vivo* efficacy validation in appropriate animal models of colorectal cancer. The mechanism of action suggests the potential for combination therapies with conventional chemotherapeutics, possibly overcoming resistance mechanisms mediated by CLIC1-dependent survival pathways. Biomarker development, including CLIC1 expression levels, subcellular localization, and functional readouts, will be essential for patient stratification and therapeutic monitoring in clinical trials targeting CLICs.

In conclusion, this work establishes a proof-of-concept for pharmacological targeting of CLIC1 through allosteric modulation of its conformational dynamics, providing the first validated small-molecule inhibitor of CLIC1 membrane translocation. The discovery and mechanistic characterization of NSC602247 lay the groundwork for next-generation therapeutics against colorectal cancer. Overall, our results define a new paradigm for drugging proteins whose functions are dictated by conformational switches and cryptic pockets, expanding the scope of tractable targets for cancer therapy.

## Experimental procedures

### Computational methods

#### MixMD simulations

All MixMD simulations were performed using Desmond (Maestro 2020-2, Schrödinger) with the HsCLIC1 crystal structure (PDB: 1K0M) as the starting model. The structure was prepared by removing water molecules, adding explicit hydrogens, and setting protonation states at pH 7.4 ± 0.5. Hydrogen bond optimization was conducted with ProtAssign, followed by restrained minimization using the OPLS3e force field. The protein was solvated in an orthorhombic TIP3P water box with 10 Å buffer, and probe molecules (acetonitrile, isopropanol, and pyrimidine) were added at 5% (v/v) for each simulation. Sodium and chloride ions were added to reach a final concentration of 150 mM.

System relaxation and equilibration consisted of a series of six steps with restraints on the solute: two initial Brownian dynamics NVT stages (simulation performed under constant number of particles, volume, and temperature) at 10 K for 100 ps and 12 ps, followed by two NPT equilibration (simulation performed under constant number of particles, pressure, and temperature) stages at 10 K for 12 ps each with heavy atom restraints. The pocket was then solvated, followed by two short NPT equilibrations for 12 ps and 24 ps. The simulations used RESPA integration with a 2 fs timestep and a 9 Å cutoff for nonbonded interactions. The NPT ensemble was maintained using a Nosé–Hoover chain thermostat and Martyna–Tobias–Klein barostat. After equilibration, ten independent 20 ns production runs were performed per probe, totaling 350 ns per probe and 1050 ns overall. Probe occupancy and pocket analysis were conducted using ProbeView (Schrödinger).

#### Molecular docking and virtual screening and MD simulations

Molecular docking and virtual screening were performed using the cryptic binding site identified on HsCLIC1 as the target. The National Cancer Institute -enhanced compound library was screened using grid-based docking with GLIDE (Schrödinger Suite), which applies constraints favoring interactions with key hotspot residues. The top-ranked compounds were further refined using MM-GBSA calculations to estimate the binding energy and solvation effects.

Following docking, MD simulations were carried out to assess the stability of the HsCLIC1-ligand complexes. Systems, including both the HsCLIC1-NSC602247 complex and apo HsCLIC1 as a control, were prepared with the Protein Preparation Wizard (Schrödinger) by assigning protonation states at pH 7.4 ± 0.5, adding explicit hydrogens, and optimizing hydrogen bonds using ProtAssign. Restrained minimization was performed with the OPLS3e force field. The complexes were solvated in orthorhombic boxes with TIP3P water extending 10 Å from the protein surface. Counterions were added to neutralize the charge, with a final salt concentration of 0.15 M NaCl. The systems underwent multistep energy minimization and equilibration protocols involving Brownian dynamics and NPT ensembles at 10 K with restrained solute atoms, followed by gradual relaxation and solvation of the binding pocket. Subsequently, 1 μs production MD simulations were run in Desmond under NPT conditions using RESPA integration (2 fs timestep) with a 9 Å cutoff for nonbonded interactions. The temperature and pressure were controlled using the Nosé–Hoover chain thermostat and Martyna–Tobias–Klein barostat. The trajectory data were saved every 50 ps.

Analyses, including root mean square deviation, Rg, and solvent accessible surface area, were performed using simulation event analysis tools in Maestro. The ligand-bound and apo states were compared to assess their stability and conformational changes.

##### Binding free energy calculation

The binding free energy (ΔG_bind_) was calculated to assess the stability and affinity of NSC602247 at the dimer interface of HsCLIC1. For this purpose, snapshots were extracted at 1 ns intervals from the 1000 ns MD trajectory of the HsCLIC1–NSC602247 complex. MM-GBSA binding energy calculations were performed for each frame using the Prime MM-GBSA module (Schrödinger 2020-2) and the VSGB 2.0 solvation model (57). The binding free energy for each frame was calculated as:

$$\Delta G_{\text{bind}}=G_{\text{complex}}-\left(G_{\text{receptor}}+{\;G}_{\text{ligand}}\right)$$

where G_complex,_ G_receptor_, and *G*_*ligand*_ are the absolute free energies of the HsCLIC1- NSC602247 complex, the receptor (HsCLIC1), and NSC602247 alone, respectively. A more negative value indicates stronger binding, as the MM-GBSA value denotes the total free energy of binding. The total ΔG energy was calculated as the sum of all the interactions established between the protein and ligand throughout the simulation. It generates a file containing the breakdown of various interactions at the binding site. Using this file, we ran the “Energy decomposition” script (https://www.schrodinger.com/scriptcenter) and estimated the energies of each interacting residues and their types of interactions, such as hydrophobic interactions (π-π and π-cation interactions), solvation effects, hydrogen bonding, van der wall interactions and charged coulombic interactions. All the simulations, calculations, and analyses were performed on the Prime Module of Schrodinger 2020-2.

#### Mutational analysis in silico

*In silico* mutational analysis was performed to evaluate the contribution of key interfacial residues. The hotspot residues Tyr214 (Site 1) and Phe83 (Site 2), which are responsible for π–π interactions, were mutated to alanine using Maestro 2020-2 (Schrödinger). The mutated HsCLIC1–NSC602247 complex was prepared as described above, solvated with TIP3P water and 0.15 M NaCl, and minimized. MD simulations for the mutant complex were performed under NPT conditions using the OPLS3e force field at 300 K and 1.0315 bar for 1000 ns. The aim was to assess the stability of the complex and the impact of mutations on ligand binding and dimer integrity.

### Protein expression and purification

Human HsCLIC1 was cloned and inserted into pETM30 (a kind gift from EMBL) and expressed in BL21(DE3) *E. coli* cells following an in-house protocol (8, 58). Briefly, cultures were grown in LB medium with 50 μg/ml kanamycin at 37 °C to an OD_600_ of 0.6, and then, the cells were induced with 1 mM IPTG at 25 °C for 16 h. The cells were harvested, resuspended, and lysed by sonication in lysis buffer (50 mM Tris-HCl pH 8.0, 150 mM NaCl, 10 mM imidazole, 10 mM MgCl_2_, 1 mM benzamidine, 1 mM PMSF, and 3 mM β-mercaptoethanol). The lysate was clarified at 13,000 to 15,000×*g* for 45 min, filtered, and loaded onto HisTrap FF columns (2 × 5 ml) preequilibrated with buffer A (50 mM Tris-HCl pH 8.0, 150 mM NaCl, and 10 mM imidazole). After washing, the protein was eluted on an increasing gradient of buffer B (50 mM Tris-HCl pH 8.0, 150 mM NaCl, 500 mM imidazole). The peak fractions were pooled, dialyzed overnight with TEV protease, and further purified using size exclusion chromatography (Superdex S75 16/600, Cytiva) in 10 mM Tris-HCl (pH 8.0) and 150 mM NaCl (with or without 2 mM DTT). Protein concentrations were determined by A_280_ using the theoretical extinction coefficient. The protein was concentrated to 25 mg/ml, flash frozen in LN2 and stored at −80 °C for long-term storage.

### Site-directed mutagenesis

The Y214A substitution in HsCLIC1 was generated by site-directed mutagenesis to investigate the role of Tyr214 in ligand binding. Mutagenic PCR was performed using complementary primers containing the desired mutation (Table S1) and pETM30-HsCLIC1 as the template. Amplification was carried out with Phusion High-Fidelity DNA polymerase (New England Biolabs, Cat. #M0530S) using a two-step PCR strategy. Briefly, separate forward and reverse reactions (25 μl each) were first amplified for 10 cycles, after which the reactions were combined and subjected to an additional 20 cycles to obtain the full-length mutated plasmid. The PCR product was treated with DpnI (New England Biolabs, Cat. #R0176S) at 37 °C for 1 h to remove methylated parental DNA. The digested product was transformed into chemically competent *E*. *coli* TOP10 cells and plated on LB agar containing kanamycin. Positive colonies were screened and verified by Sanger sequencing using pGEX_5 and T7 terminator primers (Macrogen Inc.). The verified construct was designated pETM30-HsCLIC1 Y214A and used for further studies. The mutant protein was expressed and affinity purified using the same protocol as for the native protein.

### Biophysical characterization

#### Surface Plasmon Resonance (SPR)

SPR experiments were conducted using a Biacore T200 system (Cytiva). The purified HsCLIC1 protein was diluted to 50 μg/ml in 10 mM acetate buffer (pH 4.5) and immobilized on a Series S Sensor Chip CM5 (Cytiva, Cat. # BR100530) using amine coupling according to the manufacturer's instructions. Data acquisition was controlled using Biacore control software version 2.0.1 (Cytiva; https://www.cytivalifesciences.com/en/us/support/software/biacore-downloads). Immobilization was performed by passing NHS/EDC (Cytiva, Cat. # BR100050) to activate the sensor surface for 420 s followed by passing HsCLIC1, resulting in approximately 13,000 response units (RU). Then, the surface was blocked by passing ethanolamine (Cytiva, Cat. # BR100050) for 420 s at a flow rate of 10 μl/min. The running buffer consisted of 10 mM sodium phosphate (pH 7.4), 150 mM NaCl, and 1% DMSO (Sigma, Cat. #34943). NSC602247 was prepared in running buffer at concentrations ranging from 196 nM to 12.5 μM (twofold dilutions) and injected over the immobilized protein using standard multicycle kinetics with 120 s association and 120 s dissociation phases at a flow rate of 30 μl/min. Between cycles, the protein was regenerated by addition of 10 mM glycine (pH 2.5) for 30 s at a flow rate of 30 μl/min. The data were analyzed with Biacore Evaluation Software 3.1, and equilibrium dissociation constants (*K*_*D*_) were calculated using steady-state affinity fitting. SPR experiments were performed in triplicates, and the *K*_*D*_ is presented as the mean average with standard deviation. n = 3.

#### Microscale thermophoresis (MST)

HsCLIC1 and HsCLIC1-Y214A mutant were labeled using the RED-NHS Kit (NanoTemper, Cat. #MO-K022). 20 micromolar HsCLIC1 was incubated with 60 μM dye in labeling buffer according to the manufacturer’s protocol (molar ratio; dye:protein:3:1) for 30 min at room temperature in the dark. The unreacted dye was removed by desalting Microspin G25 columns (Cytiva, Cat. #27532501) equilibrated with PBS (pH 7.4). The degree of labeling was assessed by UV/Vis spectrophotometry at 650 and 280 nm, yielding a typical labeling efficiency of 0.8. Both proteins were diluted to 400 nM in PBS. NSC602247 was prepared in PBS (pH 7.4) and serially diluted (1:1) across 12 concentrations, yielding concentrations ranging from 400 μM to 195 nM. For MST measurements, equal volumes of each NSC602247 dilution and labeled protein mixture were mixed, resulting in final concentrations of 200 nM HsCLIC1 and 200 μM to 97.5 nM NSC602247. The samples were incubated for 10 min at room temperature and centrifuged at 10,000×*g* for 10 min. Subsequently, the samples were loaded into standard MST capillaries (NanoTemper Cat. #MO-K022).

Measurements were performed on a Monolith NT.115 instrument (NanoTemper Technologies, 24 channels) at 25 °C, with instrument settings of 40% LED power and medium MST power. Data from three independent replicates were analyzed using MO.Affinity Analysis 3 (NanoTemper Technologies; https://shop.nanotempertech.com/mo-affinity-analysis-3-software-unlimited-licenses/) software focused on the signal at an MST-On time of 1.5 s. The change in normalized fluorescence (Δ*F*_*norm*_, ‰) was plotted against the ligand concentration to generate binding curves and calculate the dissociation constant (*K*_*D*_).

$$\Delta F_{\text{norm}}={\;F}_{\text{hot}}/F_{\text{cold}}\times 1000$$

where *F*_*cold*_ represents fluorescence before IR heating and where *F*_*hot*_ represents fluorescence after heating. Experiments were performed in triplicates, and the *K*_*D*_ is presented as the mean average with standard deviation. n = 3 (Table S2).

### DLS

PBS buffer (pH 7.4) was filtered through a 0.02 μm Whatman Anotop 10 filter (Cytiva, Cat. #6809-1102) prior to sample preparation. Stock solutions of native CLIC1 and Y214A mutant (25 mg/ml) were centrifuged at 16,000×*g* for 45 min to remove aggregates, and the clarified supernatant was collected and diluted to a final concentration of 2.5 mg/ml (92.5 μM) in filtered PBS. For small-molecule experiments, protein samples (2.5 mg/ml) were incubated with 200 μM NSC602247 in PBS for 10 min on ice. A 50 μl aliquot of each sample was transferred into a low-volume quartz cuvette (Hellma Analytics, Cat. #105.252-QS). The cuvette was thoroughly cleaned with methanol, ethanol, and Milli-Q water between measurements. DLS measurements were performed at 25 °C using a Malvern Zetasizer Nano S (Malvern Panalytical Ltd; htps://www.malvernpanalytical.com/en/support/product-support/software/zetasizer-nano-software-update-v3-30). Each sample was measured in triplicate, and data were analyzed using Zetasizer software (version 8.01, Malvern Panalytical Ltd; https://www.malvernpanalytical.com/en/support/product-support/software/zetasizer-nano-software-update-v3-30). The d.nm value represents hydrodynamic diameter in nanometer.

### Enzyme kinetics

The DHA reductase activity of HsCLIC1 was assayed as described by Stahl (59) with minor modifications. The reaction was initiated by adding 100 μl of 2 mM GSH to the reaction mixture, which contained 5 μM protein, 0.2 mM EDTA, and increasing concentrations (0.1 mM to 10 mM) of DHA in MES buffer, pH 5.8. The formation of ASA was monitored at OD_265_ over 10 min, with readings taken every 10 s. The initial velocity (Vo) was calculated by taking the OD_265_ until the enzyme reaction was linear and then determining the amount of ascorbate formed using a standard curve. Ascorbate formed divided by the total time of linear ascorbate formation gives the V_o_ in μM/min. The data were normalized to the negative control (protein heat inactivated at 60 °C for 5 min) as 0% enzyme activity, while the positive control (without any inhibitor) was treated as 100% enzyme activity. For the enzyme inhibition assays, the above reaction with the CLIC1 enzyme was supplemented with 50 μM compound, and for each DHA concentration, the reaction was repeated three times. Buffer, heat-denatured protein, and bovine serum albumin (Biobasic) were used separately as assay controls. Curve fitting and values of V_max_ and K_m_ were calculated using a nonlinear regression function with GraphPad Prism Software ver. 8.4.3; https://www.graphpad.com/scientific-software/prism/.

### X-ray crystallography

Crystallization of HsCLIC1 with NSC602247 was set up using sitting drop plates using a Mosquito robot (TTP Labtech). HsCLIC1 (25 mg/ml) was preincubated with 5 mM NSC602247 on ice for 1 h, and three drops were set with protein-to-reservoir ratios of 1:1, 1:2, and 2:1 and a final volume of 150 nl/drop. Each drop was equilibrated against 40 μl of the corresponding reservoir solution at 20°C using commercially available screens. Diffraction-quality crystals appeared in the presence of 20% PEG 6000, 0.2 M MgCl2, and 100 mM MES, pH 6.0, within 1 week. The crystals were then cryopreserved in reservoir conditions containing 20% glycerol. X-ray data were collected at home source with a Rigaku FR-E+ SuperBright microfocus rotating anode (Cu-Kα) generator and an R-AXIS IV^++^ d etector (National Institute of Immunology). A total of 360 degrees of data were collected with 1° oscillation. The data were processed and integrated using autoPROC (60).

Crystals of the HsCLIC1-Y214A mutant were grown under conditions identical to those described above for the native protein–NSC602247 complex. Diffraction data were collected in-house using a Rigaku XtaLAB Synergy Custom system equipped with an FR-X microfocus rotating anode X-ray generator (Cu-Kα radiation), a four-circle kappa goniometer, and a HyPix-6000HE detector at ICGEB, New Delhi. Data collection and processing were carried out using CrysAlisPro (Rigaku). For both the HsCLIC1–NSC602247 complex and the HsCLIC1-Y214A mutant, the structures were determined by molecular replacement using Phaser (61), followed by one round of automated model building using the AutoBuild (62) module of the PHENIX suite (63). Iterative model building was performed using Coot (64) and refined using either PHENIX or autoBUSTER (65). Figures were made using UCSF Chimera (66).

### Cell-based assays

#### Cell culture

Colo DM 320 and Colo 205 cells were maintained in RPMI 1640 (HIMEDIA, Cat. #AL199A) supplemented with 10% FBS (GIBCO, Cat. #A5256801) and 5% penicillin/streptomycin (HIMEDIA, Cat. #A018). Similarly, HT29 HCT 116 and Caco-2 cells were maintained in complete DMEM (HIMEDIA, Cat. #AL007A) with 10% FBS. All cell lines were maintained at 37 °C and 5% CO2. The cell lines were obtained from NCCS, Pune India, and validated at the source. All the cells were *Mycoplasma* tested in-house using MycoFluor *Mycoplasma* detection kit (Thermo Fisher Scientific, Cat. #M7006).

#### RT‒PCR quantification of CLIC1 expression

Real-time PCR analysis was performed to assess the gene expression profiles of CLIC isoforms (CLIC 1–6) in colorectal cancer cells. RNA was isolated from four colorectal cancer cell lines, HT29, Caco-2, Colo DM 320, HCT-116 and Colo 205, using TRIzol-based method (67). Briefly, cold TRIzol was added to the cells, and the samples were incubated at room temperature (RT) for 5 min to disrupt the cells and facilitate the complete dissociation of nucleoprotein complexes. This was followed by adding chloroform to each sample and shaking the tube vigorously for 15 s. After the samples were incubated for 5 min at RT, the samples were centrifuged at 12,000 rpm for 15 min at 4 °C. The topmost aqueous layer was separated, and isopropanol was added to it. Following an incubation period of 10 min at RT, the samples were spun down at 14,000 rpm for 25 min at 4 °C. The resulting pellet was dissolved in nuclease-free water and quantified using Nanodrop (NanoDrop 2000, Thermo Fisher Scientific). One microgram of RNA was treated with DNase I for complete removal of contaminated genomic DNA. Following the manufacturer’s protocol, the RNA prepared was reverse transcribed to cDNA using the SuperScript IV First-Strand Synthesis System (Invitrogen, Cat. #18091050). With this cDNA as a template, gene-specific primers for clic1-6 (Table S3), PowerUp SYBR Green Master Mix (Applied Biosystems, Cat. #A25741), and real-time PCR (CFX96 Touch, Bio-Rad) were used to quantify the transcript levels of CLIC 1 to 6.

#### Cell viability assay

Screening was performed with HT29 (colorectal adenocarcinoma) cell line because it showed the highest expression of CLIC1 among these cell lines. The cells (∼7000 per well/100 μl) were seeded in 96-well plates and incubated in a CO_2_ incubator for 8 h to overnight in high-glucose DMEM with 10% FBS. Compound dilutions (50 μM) were prepared in high-glucose DMEM with 2% FBS in 1.5 ml tubes for each compound in triplicate. After removing the consumed media, 100 μl of the prepared compounds were added to each well of the seeded cells. The cells were allowed to grow in the presence of a compound for 48 h. After 48 h incubation, sterile MTT (3-(4,5-dimethylthiazol-2-yl)-2,5-diphenyltetrazolium bromide) solution was added at a final concentration of 1 mg/ml to the media and further incubated for 4 h in a CO2 incubator. After the incubation, plate was centrifuged at 800×*g* for 5 min, the supernatant was carefully removed, 100 μl of 100% DMSO was added to the well, and the mixture was shaken for 10 to 15 min. Absorbance was measured at 550 nm within 1 h of the addition of DMSO. The absorbance values were normalized to those of the vehicle-control (0.1% DMSO) wells, which were set to 100% viability. All the analyses were done, and figures were made using GraphPad Prism 8.4.3.

#### Flow cytometry

Membrane and total (membrane and cytoplasmic) HsCLIC1 were detected using flow cytometry with an HsCLIC1-specific primary antibody conjugated with Alexa Fluor 488 (Invitrogen). For this purpose, HT29 cells were seeded at a density of 0.3 × 10^6^ cells per well in a 6-well plate and allowed to attach overnight. The cells were treated with 10 μM NSC602247 for 48 h along with 0.1% DMSO for the vehicle control and untreated controls, after which the cells were harvested, washed with PBS, and transferred to round-bottom 96-well plates for immunostaining. In brief, the cells were incubated with an Fc receptor blocker for 10 min and then stained with a custom rabbit polyclonal anti-HsCLIC1 antibody (prepared in-house, validated by Western blotting) conjugated to Alexa Fluor 488 (Invitrogen) for 1 h before being washed three times with PBS and fixed with 4% formaldehyde. For detection of cytoplasmic HsCLIC1 (in addition to membrane HsCLIC1), the cells were permeabilized with 0.1% Triton X-100 for 10 min at room temperature before being stained with a primary antibody. All intermediary centrifugation steps were performed at 2000×*g* for 5 min at 4 °C. The stained and fixed HT29 cells were analyzed using flow cytometry (FACSCanto II, BD Biosciences), and fluorescence intensities were quantified to assess HsCLIC1 expression under different treatment conditions.

## Data availability

Coordinates and structure factors are available from PBD:7FBQ and 24UG, Raw images of X-ray diffraction data are available from https://proteindiffraction.org/.

## Statistical analysis

All data are presented as mean value ± standard error of mean. Statistical analyses (one-way ANOVAs) were conducted in GraphPad Prism, with Dunnett’s *post hoc* and Tukey’s *post hoc* tests for comparisons. “∗∗∗∗” signifies *p* < 0.0001.

## Supporting information

This article contains supporting information.

## Conflict of interest

A. G. is currently an Editor with Elsevier on different journal(s) and had no role in the editorial assessment of this manuscript. The research work presented here was done by her prior to joining her current editorial role. The other authors declare that they have no conflicts of interest with the contents of this article.
