# Supplementary material for: A cryptic-site ligand stabilizes a non-canonical interface and blocks membrane insertion of the chloride intracellular channel CLIC1
Source: J Biol Chem. 2026 May 6;302(6):113113. doi: 10.1016/j.jbc.2026.113113 (PMC13253073; doi:10.1016/j.jbc.2026.113113)
Supplement: Supplementary Files [file mmc1.pdf]

Title: A cryptic-site ligand stabilizes a non-canonical interface and blocks membrane insertion of the chloride intracellular channel CLIC1

Authors: Shobhan Kuila[1‡], Archita Ghoshal[1‡], Sibasis Sahoo[1], Muthusankar Aathi[1†], Mohd Azeem Khan[1], Love Panchariya[1], Kirti Shila Sonkar[1], Wajahat Ali Khan[1], Jaswanth Raj Pandiramesh[1], Anmol Chandele[2], Arulandu Arockiasamy[1]*

Affiliations

[1]Structural Biology Group, International Centre for Genetic Engineering and Biotechnology, Aruna Asaf Ali Marg, New Delhi 110067. India.

[2]ICGEB-Emory Vaccine Centre, International Centre for Genetic Engineering and Biotechnology, Aruna Asaf Ali Marg, New Delhi 110067. India.

[†]Present Address: School of Engineering, Ajeenkya DY Patil University, Charholi Budruk, Pune – 412105, India.

[‡]Contributed equally to this manuscript

*Correspondence should be addressed to: arockiasamy.arulandu@icgeb.org

Running title: Drugging the cryptic site of CLIC1 blocks membrane insertion

For communication:

Arockiasamy Arulandu PhD

Structural Biology Group,

International Centre for Genetic Engineering and Biotechnology,

Aruna Asaf Ali Marg, New Delhi-110067. India.

Phone: +91-11-26741358 Ext-172, Mobile: +91-9711055502

E-mail: arockiasamy.arulandu@icgeb.org

Supplementary Table 1. Site directed mutagenesis 5' phosphorylated primers

Primer Name	Primer 5'-3' Sequence	GC%	Tm
Y214A_CLIC1_FP	gtacttgagcaatgcc**GCC**gcccgggaagaattc	56%	~67°C
Y214A_CLIC1_RP	gaattcttcccgggc**GGC**ggcattgctcaagtac	56%	~67°C

Supplementary Table 2. MST results for NSC602247 with CLIC1-Native

Measurement	KD (M)	Response amplitude	Signal to noise	Standard error
**1**	2.82E-06	49.82	7.1394714	7.7157954
**2**	8.12E-06	17.80	5.2190259	4.001488
**3**	5.82E-06	17.00	5.7851562	3.2857665
**Average**	**5.59E-06**			
**Std. deviation**	**2.65E-06**			

Supplementary Table 3. Gene-specific PCR primers for CLIC1-6

Primer name	Primer sequence	Harvard primer bank ID-	Amplicon size	Tm
CLIC1_F	ACCGCAGGTCGAATTGTTC	48375182c1	123	60.4
CLIC1_R	ACGGTGGTAACATTGAAGGTG	48375182c1		60.2
CLIC2_F	AATCCTCCGTTCCTGGTGTAT	66346732c2	124	60.3
CLIC2_R	AAGACTCCTTGTACTTGGGACT	66346732c2		60.1
CLIC3_F	CCTCAAGGGCGTACCTTTCAC	40288289c1	112	62.7
CLIC3_R	GTCGCTGTCATAGAGCAGGA	40288289c1		61.1
CLIC4_F	AGTGTGACGACTGTTGACCTG	209870110c2	107	61.9
CLIC4_R	ACATCCGTTTTGACTTCACTGT	209870110c2		60.1
CLIC5_F	CTTGACCCCTGAAAAGTACCC	166197661c2	80	60
CLIC5_R	ACTTGGAAAAGATGTCGATGCC	166197661c2		60.9
CLIC6_F2	GGGACCCAACATCCCGAATC	27894377c2	161	62.3
CLIC6_R2	TCAGGCAGAGGGCTATTTAAGT	27894377c2		60.6

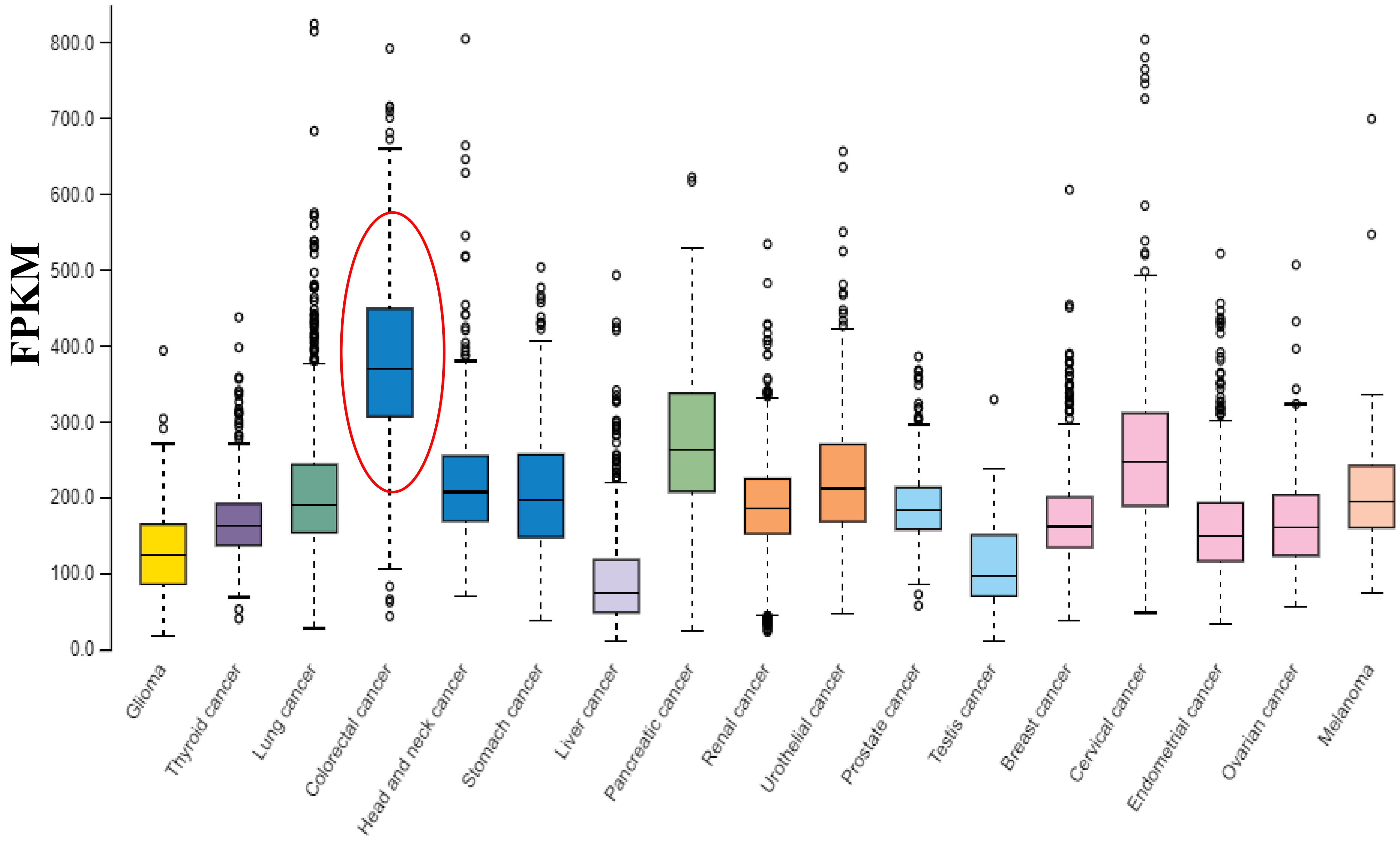

Supplementary Figure 1. CLIC1 expression in various cancers. a) TCGA data showing CLIC1 RNA expression in various cancers. The X-axis shows fragments per kilobase of transcript per million mapped fragments (FPKM). It shows highest expression of CLIC1 in colon cancer. Credit: Protein Atlas.

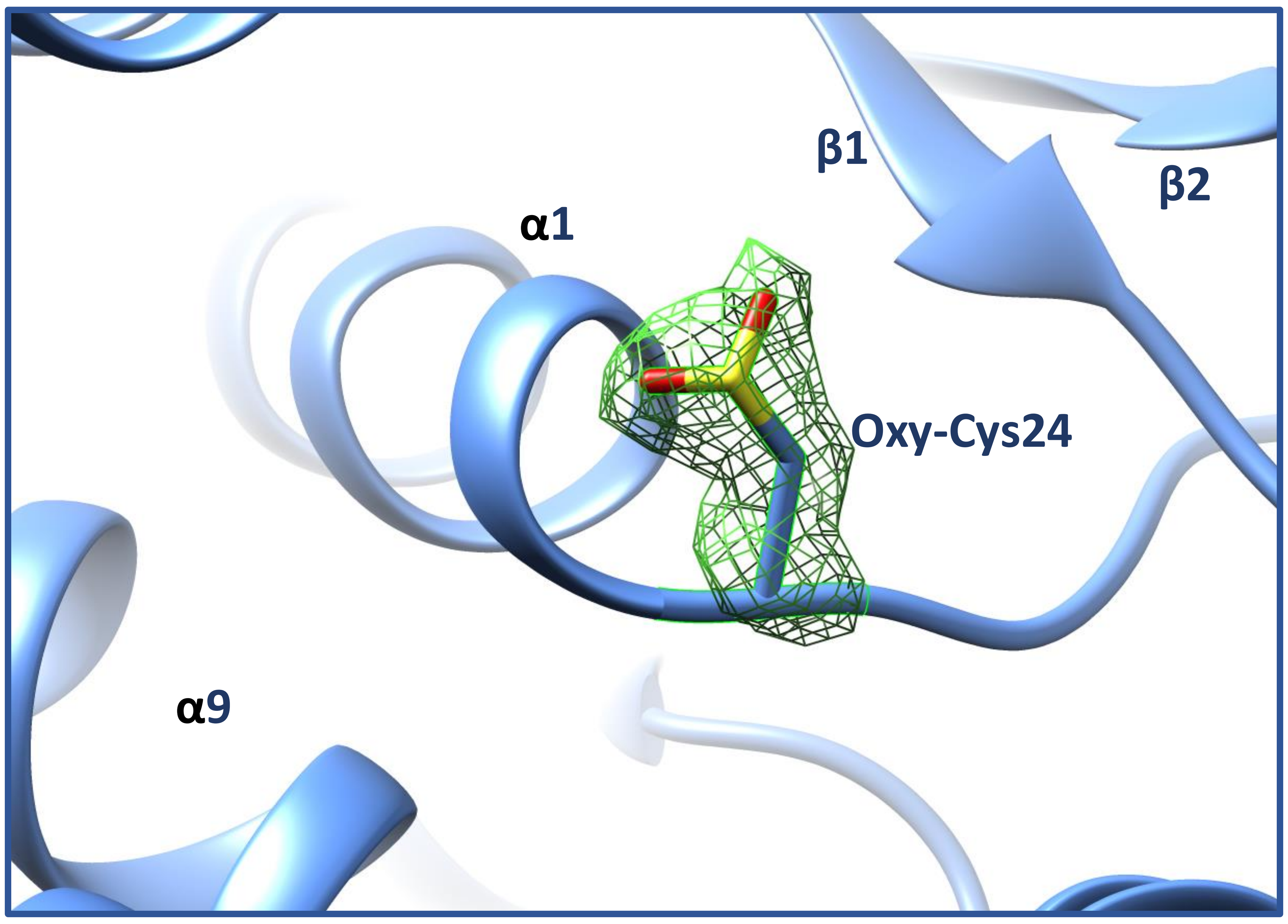

Supplementary Figure 2. Oxidation of Cysteine in crystal structure: Oxidized Sulfinic form of Cysteine (CYS24) due to crystallization artifact in crystal structure of HsCLIC1/NSC602247 complex (PDB:7FBQ)

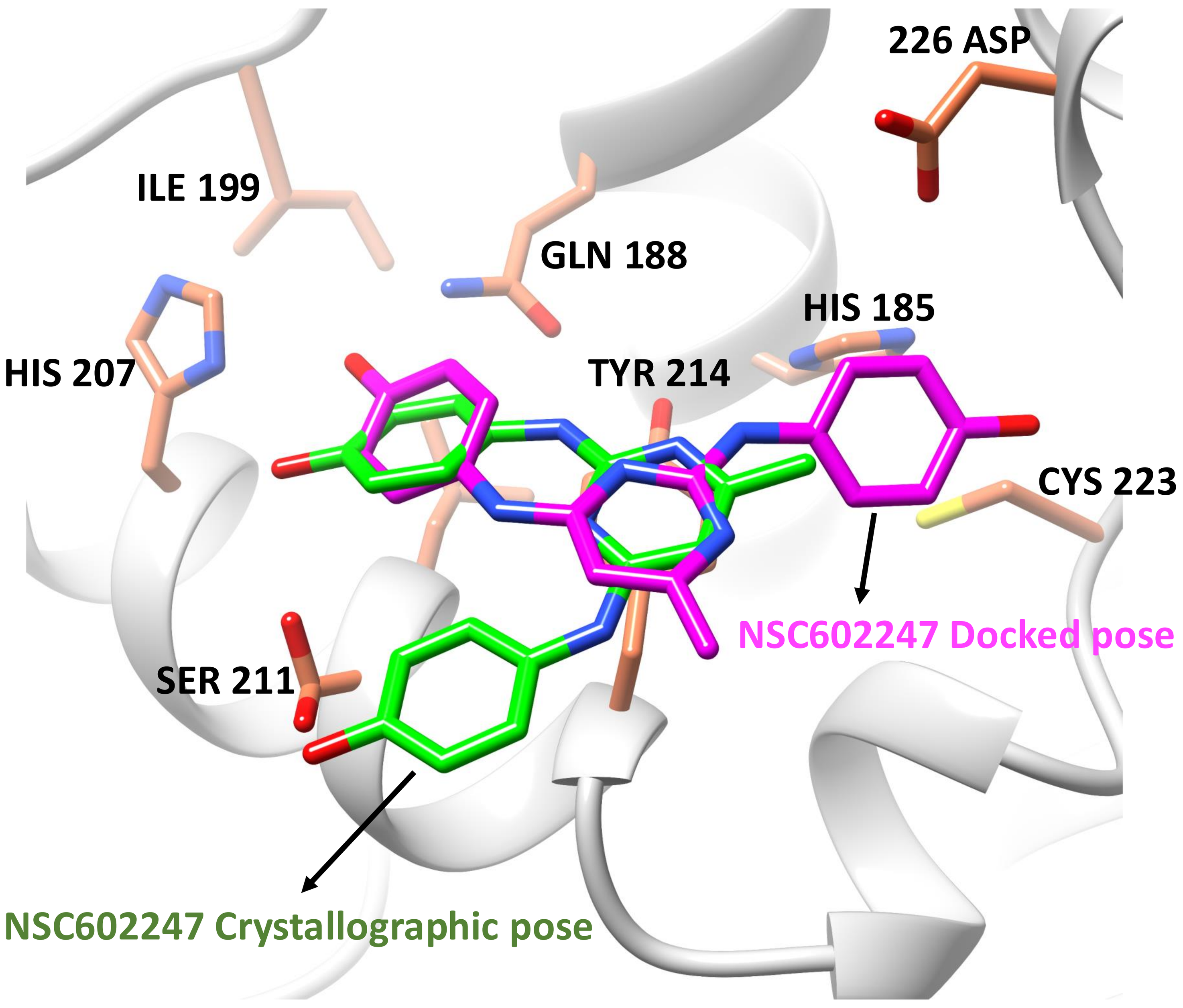

Supplementary Figure 3. Comparison of the predicted docking pose and crystallographic binding mode of NSC602247 in the CLIC1 binding pocket. The docked pose obtained from molecular docking is shown in magenta, while the experimentally determined crystallographic pose is shown in green.

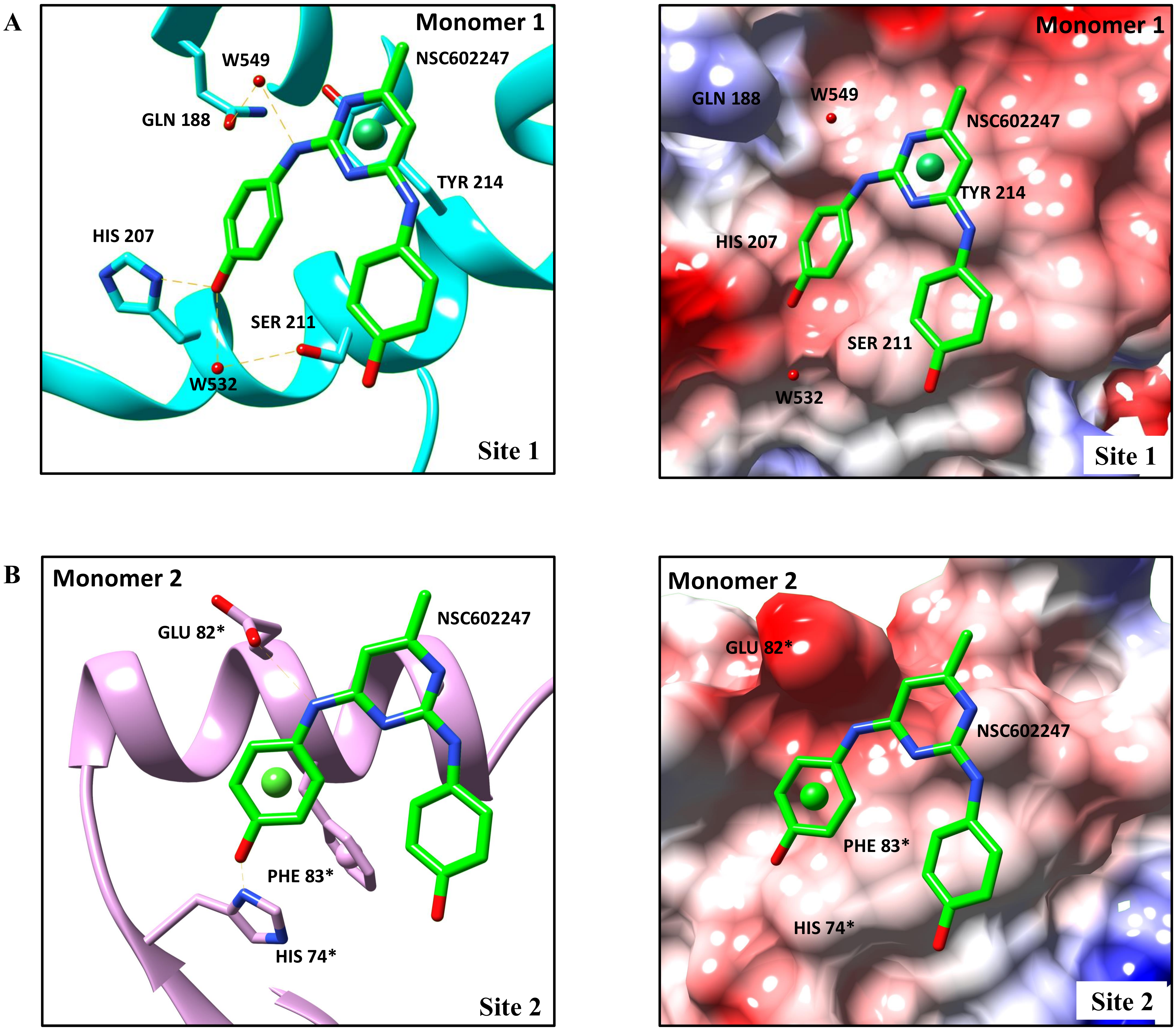

Supplementary Figure 4: Two binding sites of NSC602247 on HsCLIC1 identified by X-ray crystallography. (A) Ribbon/stick and electrostatic surface representation of the cryptic site (Site 1) on monomer 1. **(B)** Ribbon/stick and electrostatic surface representation of Site 2 at the HsCLIC1 dimer interface on monomer 2.

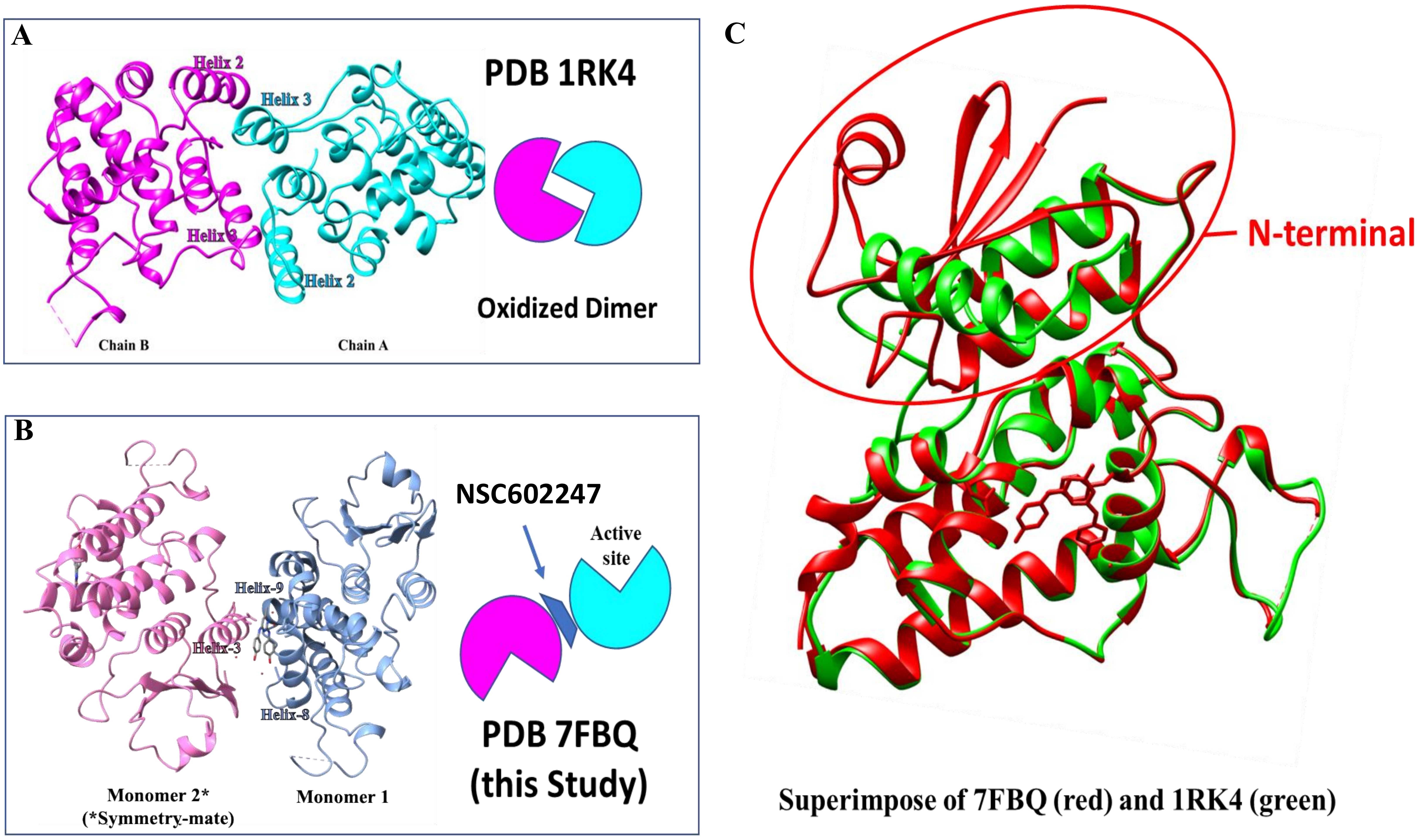

Supplementary Figure 5. Ligand-induced oligomerization differs from known HsCLIC1 dimers. A) Shows the oxidized dimer of CLIC1 (PDB:1RK4) from Littler et al., 2010. **B)** shows NSC602247 induced dimer of CLIC1 from this study (PDB:7FBQ). **C)** Superimposition of oxidized CLIC1 dimer on NSC602247 induced dimer shows that the N-terminal conformation change, proposed to be a pre-membrane inserted state, is absent in the NSC602247 induced dimer.

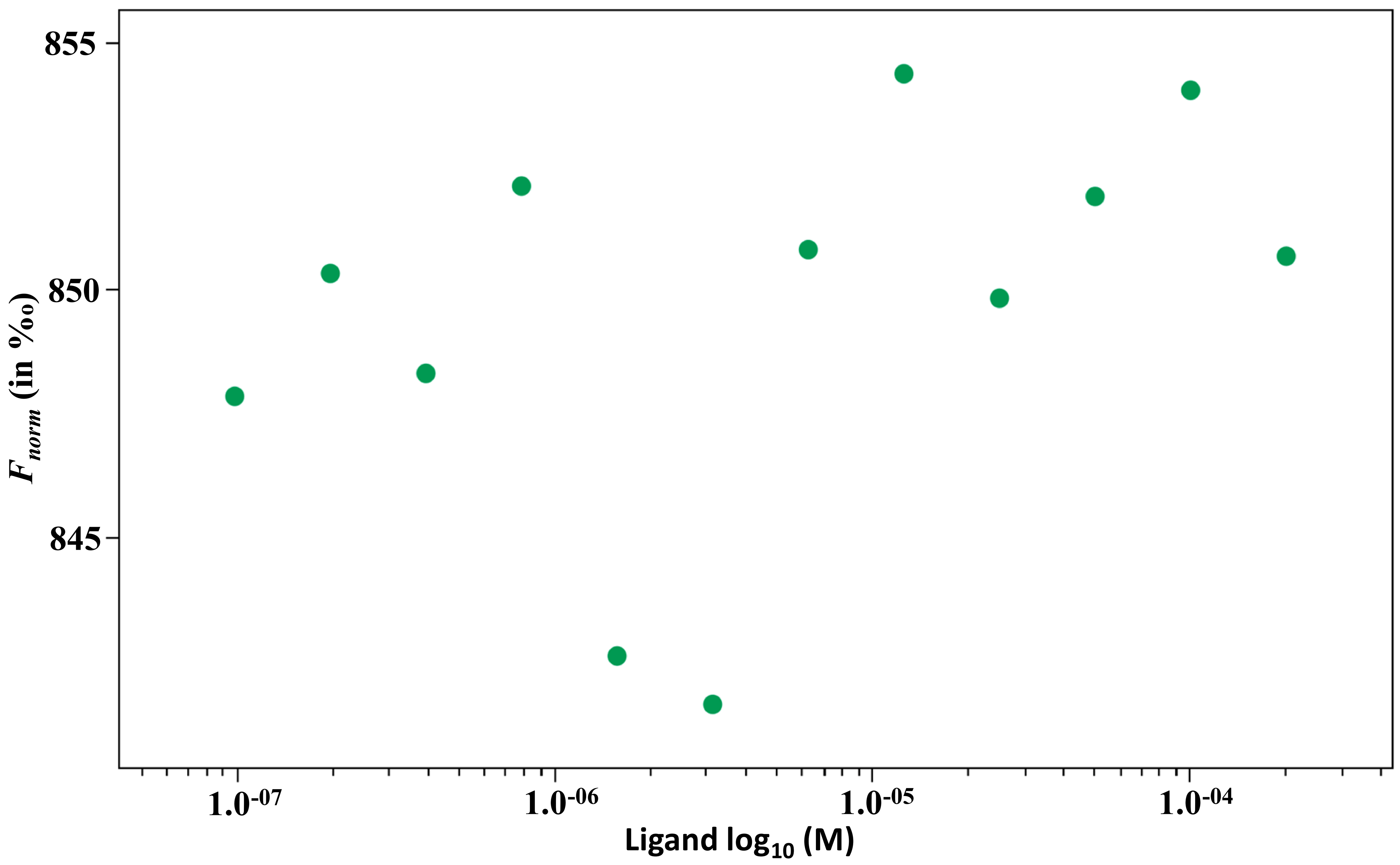

Supplementary Figure 6: MST data showing loss of ligand binding upon Y214A mutation: CLIC1 Y214A mutant showed minimal fluorescence change across the ligand concentration range, indicating a lack of detectable binding and preventing accurate KD determination using the analysis software. The experiments were done in triplicates.

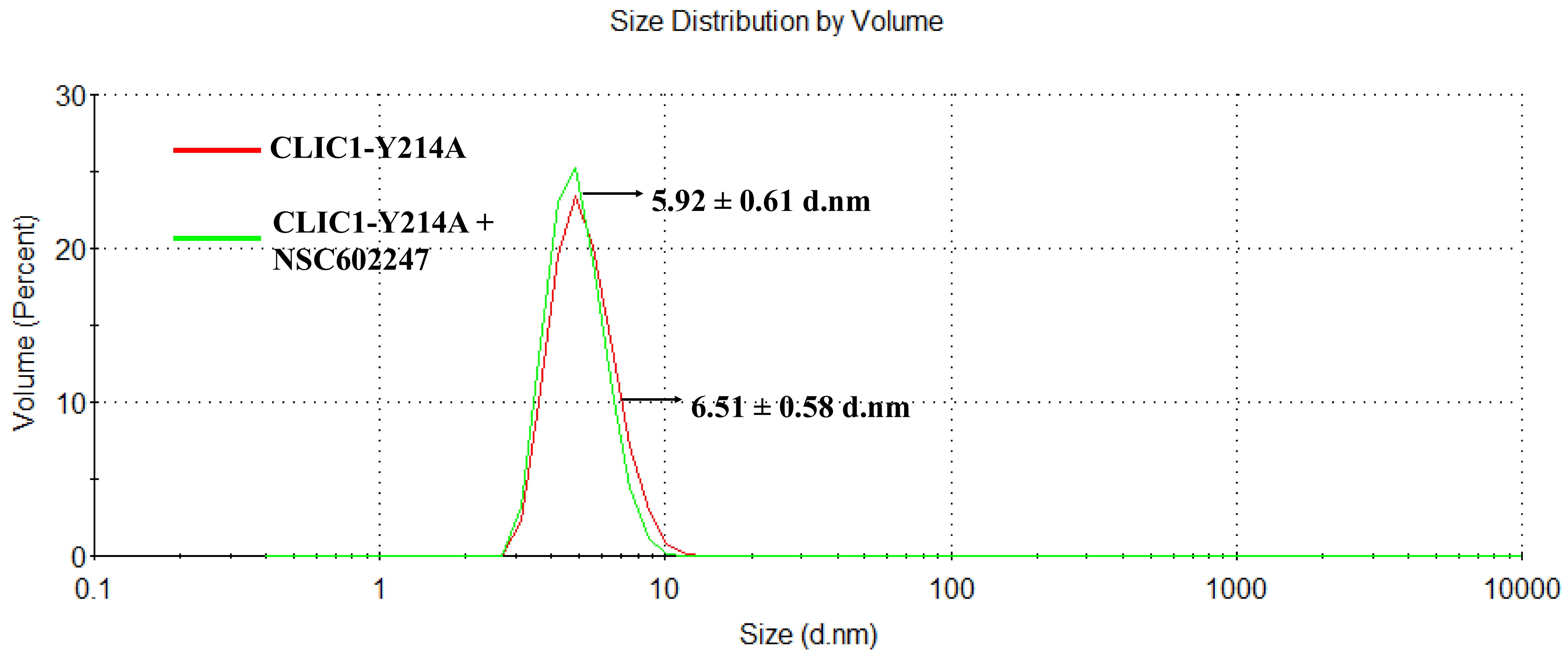

Supplementary Figure 7: Dynamic light scattering (DLS) analysis of CLIC1-Y214A in solution. Z-average hydrodynamic diameter (d.nm) of CLIC1-Y214A measured in the absence (red) and presence (green) of NSC602247.

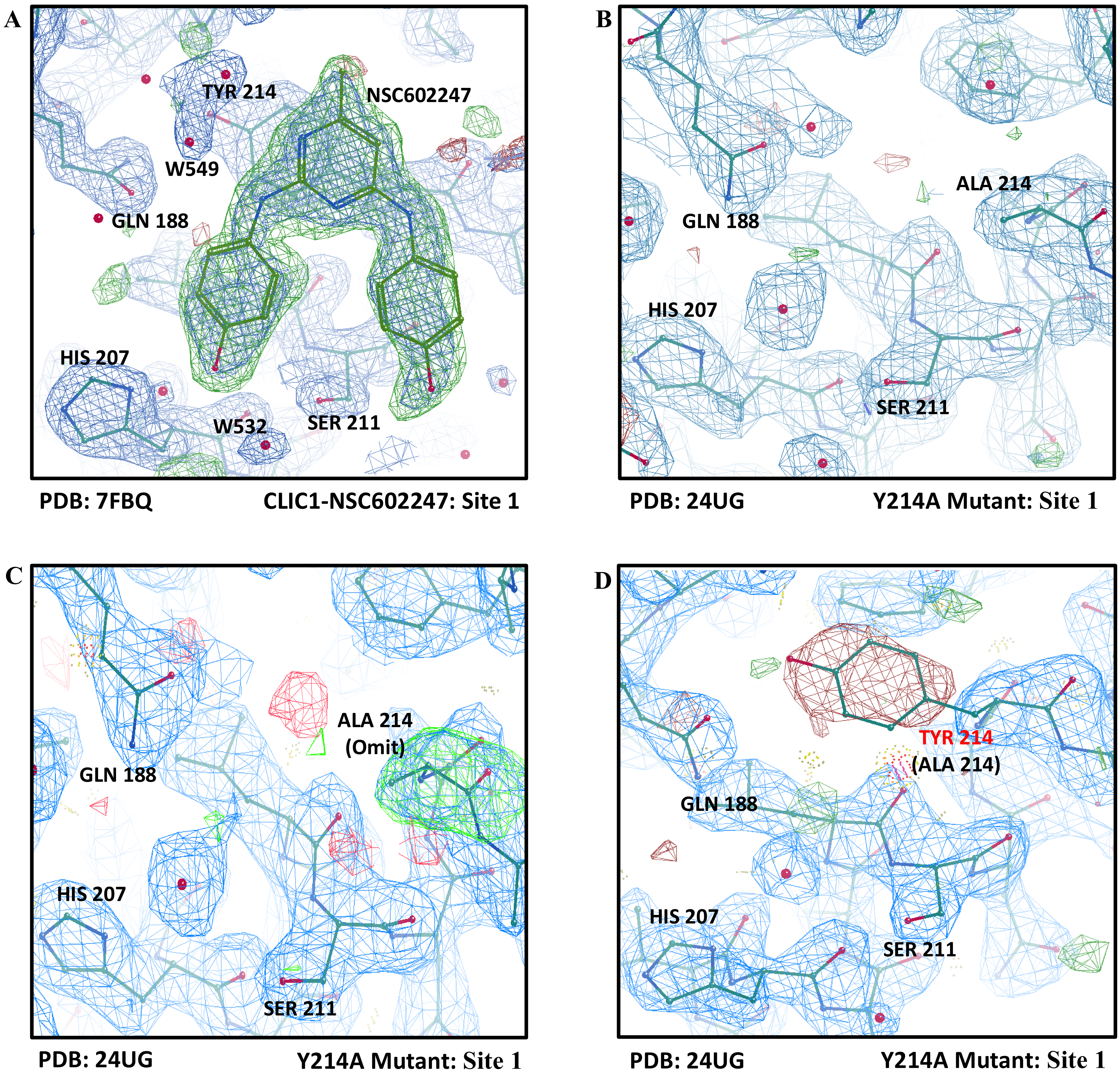

Supplementary Figure 8: Tyr214 is required for NSC602247 binding at the CLIC1 non-canonical site. A) Native CLIC1 co-crystallized with 5 mM NSC602247, in $P2_12_12_1$ space group, with one molecule per asymmetric unit (ASU), shows clear *Fo-Fc* electron density (green mesh) for the ligand at the non-canonical binding pocket (Site 1). The ligand is surrounded by residues Gln188, His207, Ser211, and Tyr214, along with nearby ordered water molecules (W532 and W549). The blue density depicts *2Fo-Fc* map contoured at 1σ. **B)** The Y214A mutant, crystallized under identical conditions, in $P2_1$ space group with two molecules per ASU, shows no detectable *Fo-Fc* electron density for NSC602247 at the same interfacial site, indicating loss of ligand binding upon substitution of Tyr214 with alanine. **C)** For additional validation, *Fo-Fc* omit difference map (with Ala214 omitted during refinement) is shown, and **D)** *Fo-Fc* difference map is shown with negative (red) density for Tyr214 when modelled in place of Ala214 is shown. Difference maps shown are contoured at 3σ. Figures were made in COOT.

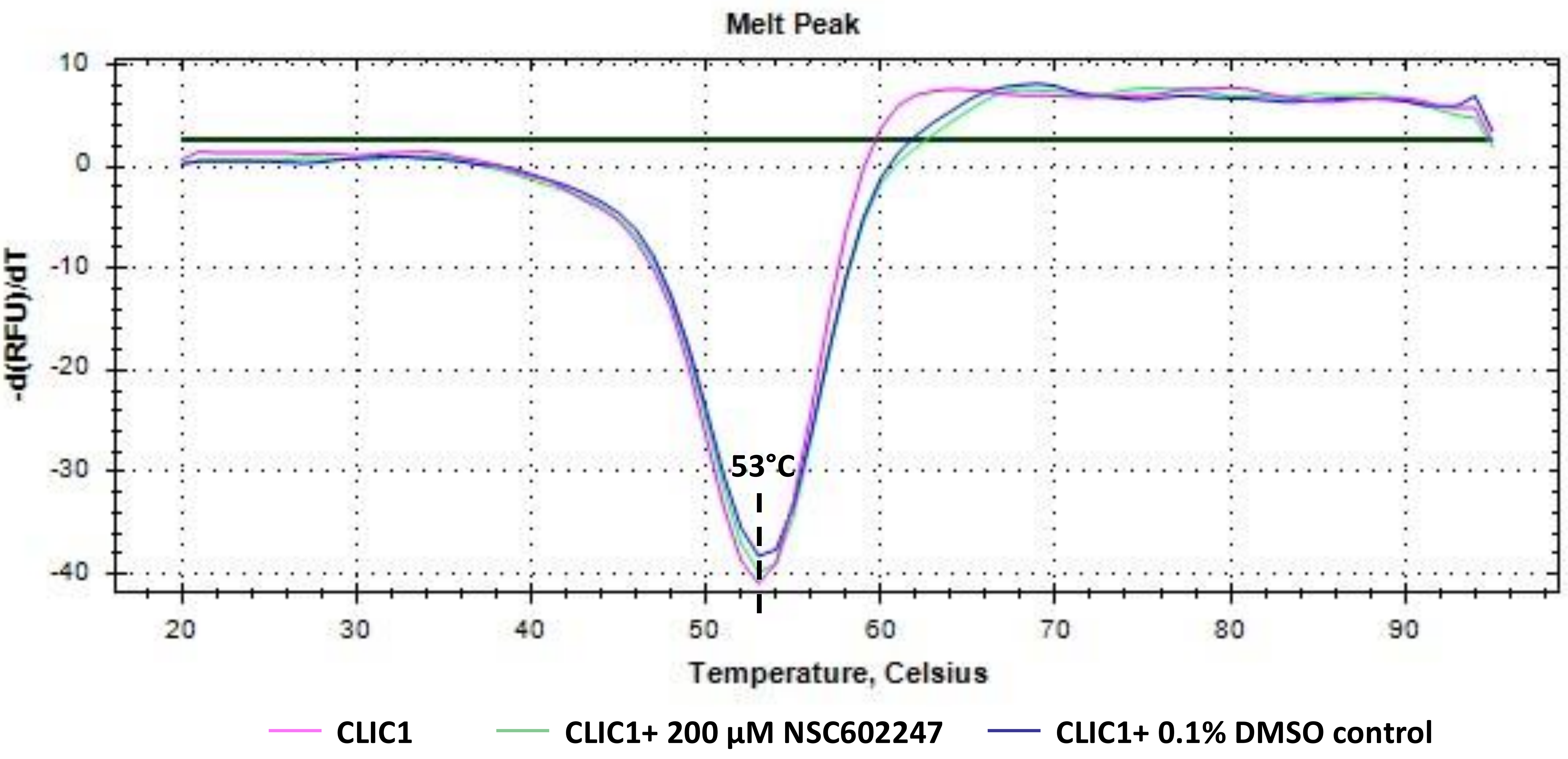

Supplementary Figure 9. Thermal shift assay (TSA) of CLIC1 with NSC602247: No significant change in melting temperature (T_m) is observed upon ligand binding.
